# Effect of Internet-Based vs Face-to-Face Cognitive Behavioral Therapy for Adults With Obsessive-Compulsive Disorder: A Randomized Clinical Trial

**DOI:** 10.1001/jamanetworkopen.2022.1967

**Published:** 2022-03-14

**Authors:** Lina Lundström, Oskar Flygare, Erik Andersson, Jesper Enander, Matteo Bottai, Volen Z. Ivanov, Julia Boberg, Diana Pascal, David Mataix-Cols, Christian Rück

**Affiliations:** 1 Centre for Psychiatry Research, Department of Clinical Neuroscience, Karolinska Institutet, Stockholm Health Care Services, Region Stockholm, Karolinska University Hospital, Stockholm, Sweden; 2 Division of Psychology, Department of Clinical Neuroscience, Karolinska Institutet, Stockholm, Sweden; 3 Division of Biostatistics, Institute of Environmental Medicine, Karolinska Institutet, Stockholm, Sweden; 4 Child and Adolescent Psychiatry Research Center, Department of Clinical Neuroscience, Stockholm Health Care Services, Region Stockholm, Karolinska Institutet Stockholm, Sweden

## Abstract

**Importance:**

Cognitive behavioral therapy (CBT) for obsessive-compulsive disorder (OCD) is a highly specialized treatment that is in short supply worldwide.

**Objectives:**

To investigate whether both therapist-guided and unguided internet-based CBT (ICBT) are noninferior to face-to-face CBT for adults with OCD, to conduct a health economic evaluation, and to determine whether treatment effects were moderated by source of participant referral.

**Design, Setting, and Participants:**

This study is a single-blinded, noninferiority, randomized clinical trial, with a full health economic evaluation, conducted between September 2015 and January 2020, comparing therapist-guided ICBT, unguided ICBT, and individual face-to-face CBT for adults with OCD. Follow-up data were collected up to 12 months after treatment. The study was conducted at 2 specialist outpatient OCD clinics in Stockholm, Sweden. Participants included a consecutive sample of adults with a primary diagnosis of OCD, either self-referred or referred by a clinician. Data analysis was performed from June 2019 to January 2022.

**Interventions:**

Guided ICBT, unguided ICBT, and face-to-face CBT delivered over 14 weeks.

**Main Outcomes and Measures:**

The primary end point was the change in OCD symptom severity from baseline to 3-month follow-up. The noninferiority margin was 3 points on the masked assessor-rated Yale-Brown Obsessive Compulsive Scale.

**Results:**

A total of 120 participants were enrolled (80 women [67%]; mean [SD] age, 32.24 [9.64] years); 38 were randomized to the face-to-face CBT group, 42 were randomized to the guided ICBT group, and 40 were randomized to the unguided ICBT group. The mean difference between therapist-guided ICBT and face-to-face CBT at the primary end point was 2.10 points on the Yale-Brown Obsessive Compulsive Scale (90% CI, −0.41 to 4.61 points; *P* = .17), favoring face-to-face CBT, meaning that the primary noninferiority results were inconclusive. The difference between unguided ICBT and face-to-face CBT was 5.35 points (90% CI, 2.76 to 7.94 points; *P* < .001), favoring face-to-face CBT. The health economic analysis showed that both guided and unguided ICBT were cost-effective compared with face-to-face CBT. Source of referral did not moderate treatment outcome. The most common adverse events were anxiety (30 participants [25%]), depressive symptoms (20 participants [17%]), and stress (11 participants [9%]).

**Conclusions and Relevance:**

The findings of this randomized clinical trial of ICBT vs face-to-face CBT for adults with OCD do not conclusively demonstrate noninferiority. Therapist-guided ICBT could be a cost-effective alternative to in-clinic CBT for adults with OCD in scenarios where traditional CBT is not readily available; unguided ICBT is probably less efficacious but could be an alternative when providing remote clinician support is not feasible.

**Trial Registration:**

ClinicalTrials.gov Identifier: NCT02541968

## Key Points

**Question:**

Are therapist-guided, internet-based cognitive behavioral therapy (ICBT) and unguided ICBT noninferior to traditional face-to-face CBT for the treatment of obsessive-compulsive disorder (OCD)?

**Findings:**

In this noninferiority randomized clinical trial that included 120 adults with OCD, participants in all 3 treatment groups had significantly improved symptoms, but the noninferiority of ICBT could not be established. The health economic evaluation indicated that both therapist-guided and unguided ICBT were cost-effective compared with face-to-face CBT.

**Meaning:**

These findings suggest that ICBT can be a cost-effective alternative for the treatment of OCD in health care contexts where access to traditional CBT is in short supply.

## Teaser

This randomized clinical trial investigates whether unguided and therapist-guided internet-based cognitive behavioral therapy (CBT) are noninferior to face-to-face CBT for adults with obsessive-compulsive disorder.

## Introduction

Cognitive behavioral therapy (CBT) is a first-line treatment for obsessive-compulsive disorder (OCD),^1^ but only a minority of patients receive it.^2^ Therapist-guided internet-based CBT (ICBT) can increase the availability of CBT because it is more accessible, easily scalable, and requires less therapist time than face-to-face CBT.^3^ ICBT is effective compared with a waiting list or active control condition in adults with OCD,^3,4,5^ with gains sustained up to 2 years after treatment.^4^ ICBT for OCD has also shown promising results across cultures^6,7,8^ and in children and adolescents.^9,10^

This randomized clinical trial (RCT) was designed to address several critical questions that remain before ICBT for OCD can be recommended for implementation in health care. First, whether guided ICBT is noninferior to individual face-to-face CBT for OCD has not been evaluated. Second, we do not know whether ICBT is equally effective when delivered without therapist support. Third, the cost-effectiveness of ICBT vs individual face-to-face CBT for OCD needs to be evaluated to guide rational clinical service development. Finally, because most previous ICBT studies have relied on self-referred participants, it is important to establish whether the treatment outcomes can be generalized to clinic-referred participants (see the complete trial protocol in Supplement 1).

## Methods

### Design

We conducted a single-blind, noninferiority, RCT comparing therapist-guided ICBT, unguided ICBT, and individual face-to-face CBT for adults with OCD. Participants were stratified according to the source of referral (self-referred vs clinic-referred participants). Randomization sequences for self-referred and clinically referred participants were generated separately, each with an even (1:1:1) distribution among treatment groups to ensure that source of referral was balanced across treatment conditions. Individuals in both ICBT groups who did not meet response criteria^11^ at the 3-month follow-up (primary end point) were offered individual face-to-face CBT.

This RCT was approved by the Regional Ethics Board of Stockholm before the start of the trial. The trial followed the Consolidated Standards of Reporting Trials Extension (CONSORT Extension) reporting guideline for noninferiority and equivalence trials.^12^ The trial protocol has been published elsewhere^13^ and is shown in Supplement 1. Randomization and trial monitoring were performed by an independent clinical trials unit, the Karolinska Trial Alliance, following good clinical practice principles. Participants signed a written informed consent form before inclusion in the trial (eTable 1 in Supplement 2).

### Participants

Participants were enrolled consecutively. Information about the trial was sent to clinics and patient organizations and was advertised at a website and social media. Participation was by referral from general practitioners, psychiatrists, or other health care professionals, or via self-referral.^14^ The participants were assessed and treated at 2 organizationally and structurally equivalent publicly funded specialist OCD clinics in Stockholm, Sweden. After completing an online screening and a brief screening interview over the telephone, suitable participants were offered a face-to-face appointment with a psychiatrist on duty for a full psychiatric assessment. The psychiatrist administered the Mini International Neuropsychiatric Interview^15^ and the Structured Clinical Interview for *Diagnostic and Statistical Manual of Mental Disorders* (Fifth Edition)^16^ to confirm OCD diagnosis and decide on inclusion or exclusion.

Eligible participants were aged 18 years or older, had a primary diagnosis of OCD according to the *Diagnostic and Statistical Manual of Mental Disorders* (Fifth Edition) and the Mini International Neuropsychiatric Interview, and had internet access. Participants were excluded if they had received CBT for OCD (including exposure and ritual prevention [ERP]) in the last 12 months; planned to undergo psychological treatment for OCD during the trial period; made psychotropic medication changes within the last 2 months; had an organic brain disorder, bipolar disorder, psychosis, alcohol or substance dependence, autism spectrum disorder, hoarding disorder or OCD with primary hoarding symptoms, or suicidal ideation; or could not read or write in Swedish.

### Randomization and Masking

The randomization sequence was generated by Karolinska Trial Alliance, using masked block randomization (stratified by referral source) in sealed envelopes. Trial investigators (O.F. and L.L., who were not involved in assessments) opened the envelopes and assigned the participants to 1 of the 3 groups. Independent assessors (J.B., V.Z.I., D.P., and C.R.) were blinded to group allocation up to the 12-month follow-up. Group allocation was inadvertently revealed on 12 occasions, after which the participants were assessed by another independent assessor at subsequent assessment points. To verify blinding integrity, blinded assessors (J.B., V.Z.I., D.P., and C.R.) guessed each participant’s group to check whether their guesses were better than chance. Of 166 guesses at posttreatment and 3-month follow-up, 67 were correct, not statistically significantly different from the expected 33% (40.4% correct; 95% CI, 32.8%-48.2%; *P* = .05).

### Interventions

#### Individual Face-to-Face CBT

Participants received 16 sessions (90 minutes each) of individual face-to-face CBT for OCD delivered over the course of 14 weeks, according to a validated CBT protocol.^17^ Sessions were held twice weekly during the first 2 weeks and once per week for the remaining period. The treatment consisted of psychoeducation, including in vivo and imaginal ERP, a technique whereby patients are encouraged to gradually face feared stimuli while refraining from performing compulsions until anxiety subsides. The last session consisted of a relapse prevention program. Most sessions were held at the clinics, but sessions could also be scheduled outside the clinic, or in the participants’ homes.

All face-to-face sessions were audio recorded to ensure that therapists adhered to the treatment protocol. One hundred fifteen sessions (19%) were randomly selected to be assessed by 2 independent psychologists (not coauthors of this article) specialized in CBT for OCD using the Cognitive Therapy Adherence and Competence Scale^18^ (eAppendix 1 in Supplement 2).

#### Therapist-Guided ICBT

Participants received OCD-NET, a previously evaluated ICBT program for 14 weeks.^3,4,5^ The program included 10 modules, unlocked consecutively by the therapist upon completion of the homework assignment of the previous module. The main components of the treatment are psychoeducation, ERP, and relapse prevention. Therapists supported the participants through the 14 weeks via asynchronous messages, encouraging them to engage in ERP exercises and troubleshooting during treatment.

#### Unguided ICBT

The unguided ICBT was identical to the guided ICBT treatment but without any therapist support. All modules were unlocked from the start of treatment, and participants were instructed to work through the modules in consecutive order during the 14 weeks of treatment. If participants experienced technical problems during the treatment, they could contact technical support for help, and contact information to emergency psychiatric services was provided (detailed description of treatments are shown in eAppendix 2, eFigure 1, and eFigure 2 in Supplement 2).

### Therapists

Therapists were 8 licensed clinical psychologists (including L.L. and O.F.), with expertise in treating OCD both face-to-face and digitally. They received supervision from the lead author (L.L.) on request and every second week at the clinic’s own scheduled supervision hours (eTable 2 in Supplement 2).

### Outcome Measures

The primary outcome measure was the masked assessor-rated Yale-Brown Obsessive Compulsive Scale (Y-BOCS; score range, 0-40, with higher scores indicating higher severity).^19^ Clinicians practiced together on case examples to establish interrater reliability on the blinded Y-BOCS assessments. The interrater reliability in this trial was excellent, with an intraclass correlation coefficient of 0.99 (95% CI, 0.92-1.00). The Y-BOCS was administered by blinded assessors before treatment, biweekly during treatment, after treatment, and at the 3-month and 12-month follow-up appointments. The biweekly assessments during treatment were administered over the telephone, whereas the other assessments took place at the clinic. The primary end point was the 3-month follow-up.

The secondary masked assessor-rated measures were the Clinical Global Impression–Severity (CGI-S) scale and Clinical Global Impression–Improvement scale (CGI-I)^20^ and the Global Assessment of Functioning.^21^ Treatment response was defined as Y-BOCS score reduction of 35% or more and CGI-I score of 2 or lower, and remission was defined as a Y-BOCS score of 12 or lower and CGI-S score of 2 or lower.^11^

Secondary participant-rated outcome measures were the Obsessive-Compulsive Inventory,^22^ the Y-BOCS–Self-Rated,^23^ the Montgomery-Åsberg Depression Rating Scale (MADRS-S),^24^ Sheehan Disability Scale,^25^ the EuroQol 5-Dimensions,^26^ the Working Alliance Inventory–Short Form,^27^ the Insomnia Severity Index,^28^ the Trimbos and Institute of Medical Technology Assessment Cost Questionnaire for Psychiatry,^29^ and the Treatment Credibility Scale^30^ (eTable 3 and eAppendix 3 in Supplement 2).

### Safety and Adverse Events

Data on adverse events and suicidal ideation were collected by blinded assessors (J.B. and V.Z.I.) biweekly during treatment, after treatment, and at the 3-month and 12-month follow-up appointments using the Safety Monitoring Uniform Report Form^31^ and the MADRS-S. If a participant scored 4 or higher on the suicidal ideation item in MADRS-S, a structured suicide risk assessment was conducted.

### Sample Size Calculation

A bootstrap simulation with 1000 samples using individual-level data from a previous trial of therapist-guided ICBT was used to provide power estimates for the current trial.^5^ With 3 treatment groups and 8 observations per participant, we estimated that a total of 120 participants would be needed to detect a slope difference between 2 groups (ie, group 1 vs group 2 and group 1 vs group 3) of 3 points on the Y-BOCS at the 3-month follow-up with greater than 90% power. When 80 of the 120 participants had undergone treatment, deidentified Y-BOCS data without the grouping variable were shared with the Karolinska Trial Alliance, which confirmed that the model assumptions used in the power calculation were in line with our original power calculation (Supplement 1 and eAppendix 4 in Supplement 2).

### Statistical Analysis

 Data analysis was performed from June 2019 to January 2022. All outcome analyses were conducted according to the intention-to-treat principle. Mixed-effects regression analyses for repeated measures with maximum likelihood estimation were used with the assumption that data were missing at random.^32^ For the primary outcome measure, the model included fixed effects for group and time, as well as individuals’ random intercept and random slope. The interaction effect of group-by-time was used to evaluate group differences at the 3-month follow-up.

The noninferiority hypothesis was tested by comparing both ICBT programs with face-to-face CBT. Noninferiority was established when the upper limit of the Wald 90% CI for the difference between treatment conditions (ICBT estimate minus face-to-face CBT estimate) did not exceed the prespecified margin of inferiority of 3 points on the Y-BOCS.^33,34^ The noninferiority margin was decided a priori on the basis of clinical judgment and our power calculation. To evaluate whether the source of referral moderated the treatment effects, a second mixed-effects model on Y-BOCS was fitted where the source of referral was included as a covariate and the source of referral-by-time interaction as well as source of referral-by-time-by-group interaction effects were evaluated. For each continuous secondary outcome, mixed-effects linear regression models with fixed effects of time and treatment group, a random intercept, and an interaction effect of treatment group-by-time were used. CGI-S and CGI-I were analyzed with mixed-effects ordinal logistic regression with maximum likelihood estimation (for detailed description of statistical analyses, see eAppendix 4 in Supplement 2).

#### Cost-effectiveness Analysis

Health economic data were collected from health organizational (eg, therapist time), direct medical (eg, costs for health care visits), and societal (eg, indirect costs associated with sick leave) perspectives (eAppendix 5 in Supplement 2) and were analyzed in relation to outcome (response rates based on the Y-BOCS) and quality-adjusted life-years (based on the EuroQol 5-Dimensions). Costs were assessed from baseline to the 3-month follow-up and were estimated using national tariffs in Sweden, which were converted to 2020 US dollars. Mixed-effects regression analyses for repeated measures were used to evaluate changes in costs, responder rates, and quality-adjusted life-years using fixed effects for group and time, their interaction, and a random intercept, with maximum likelihood estimation. Costs in relation to effects were plotted in cost-effectiveness planes. Nonparametric bootstrapping (1000 replications) was used to estimate the difference between ICBT (guided or unguided) and individual face-to-face CBT.

#### Post Hoc Analyses

To evaluate group differences in the proportions of remitters and responders, a mixed-effects logistic regression model with maximum likelihood estimation was fitted at 3-month follow-up, with group as an independent variable (eAppendix 4 in Supplement 2). All statistical analyses were conducted using R statistical software version 4.0.2 (R Project for Statistical Computing).^35^ The 2-sided α value was set to .05. Because of the increased risk of type I error due to multiple comparisons on secondary outcomes, these findings should be considered exploratory. Scripts used for the analyses are available at the Open Science Framework.^36^

## Results

### Participants

**Figure 1.**
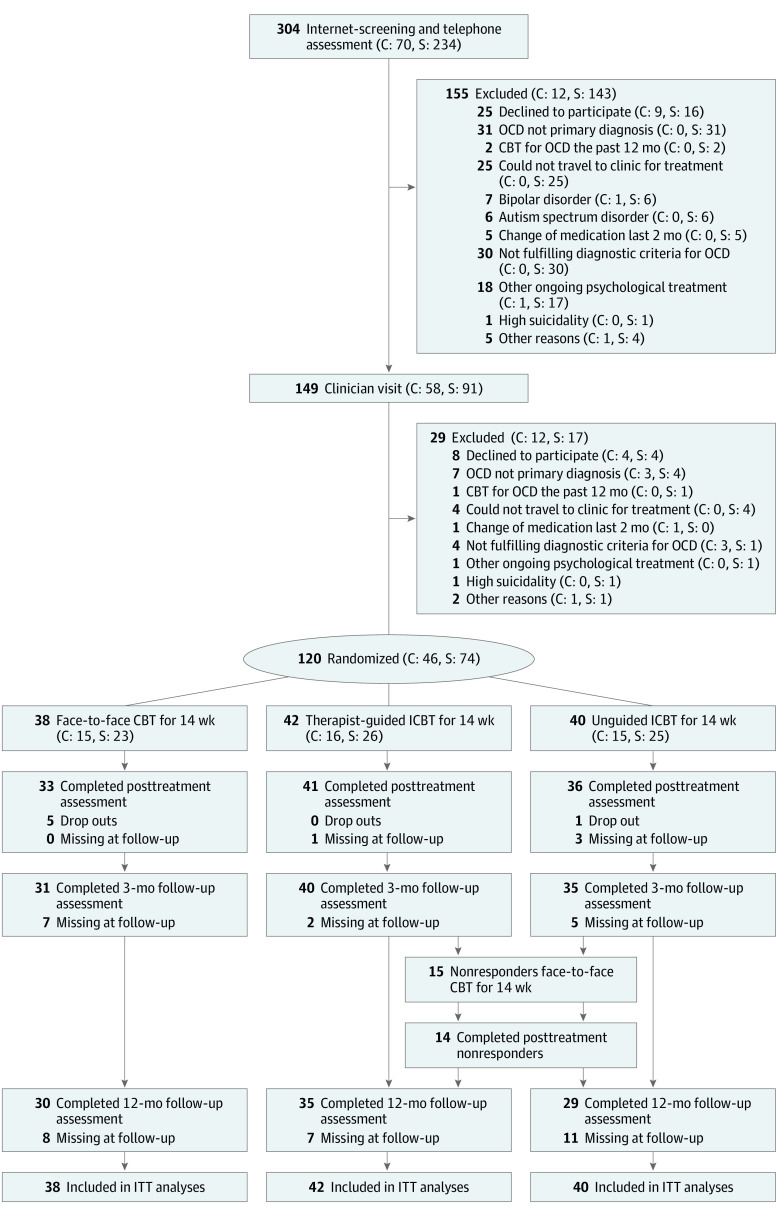
Participant Flow Through the Trial Dropouts were participants who did not complete any assessments from week 6 on. Missing at follow-up refers to participants who completed treatment but did not provide data at the follow-up time point. C indicates clinical referral; CBT, cognitive behavioral therapy; ICBT, internet-based cognitive behavioral therapy; ITT, intention to treat; OCD, obsessive-compulsive disorder; S, self-referral.

Of 304 adults screened, 120 participated in the trial between September 2015 and January 2020 (Figure 1 and eAppendix 6 in Supplement 2). Forty-six participants (38%) were clinically referred, 80 participants (67%) were women, and the mean (SD) age was 32.24 (9.64) years. Thirty-eight participants were randomized to the face-to-face CBT group, 42 were randomized to the guided ICBT group, and 40 were randomized to the unguided ICBT group. The sociodemographic and clinical characteristics of the participants are shown in Table 1 (see also eTable 4 and eAppendix 7 in Supplement 2).

**Table 1.** Participant Characteristics at Baseline

Characteristic	Participants, No. (%) (N = 120)		
	Face-to-face CBT (n = 38)	Guided ICBT (n = 42)	Unguided ICBT (n = 40)
Source of referral			
Clinical referral	15 (39.5)	16 (38.1)	15 (37.5)
Self-referral	23 (60.5)	26 (61.9)	25 (62.5)
Age, mean (SD), y	33.13 (7.93)	32.00 (9.38)	31.64 (11.46)
Sex			
Female	25 (65.8)	27 (64.3)	28 (70)
Male	13 (34.2)	15 (35.7)	12 (30)
Age of OCD onset, mean (SD), y	16.08 (8.65)	17.88 (9.68)	15.95 (7.16)
Any previous treatment for OCD	15 (39.5)	13 (31.0)	10 (25.0)
Previous suicide attempts	2 (5.0)	1 (2.0)	2 (5.0)
Source of income			
Employed	31 (81.6)	35 (83.3)	32 (80.0)
Student	3 (7.9)	6 (14.3)	5 (12.5)
Unemployed	4 (10.5)	0	3 (7.5)
Other	0	1 (2.4)	0
Level of education			
Doctorate	2 (5.3)	0	2 (5.0)
Master (>3 y university)	19 (50.0)	17 (40.5)	15 (37.5)
Bachelor (<3 y university)	6 (15.8)	9 (21.4)	10 (25.0)
High school	9 (23.7)	15 (35.7)	9 (22.5)
Primary	2 (5.3)	1 (2.4)	4 (10.0)
Main obsessions and compulsions			
Aggressive	27 (71.1)	25 (59.5)	25 (62.5)
Contamination	16 (42.1)	19 (45.2)	19 (47.5)
Unacceptable thoughts	9 (23.7)	8 (19.0)	8 (20.0)
Symmetry	10 (26.3)	9 (21.4)	8 (20.0)
Washing	15 (39.5)	18 (42.9)	18 (45.0)
Checking	28 (73.7)	27 (64.3)	28 (70.0)
Ordering	13 (34.2)	9 (21.4)	12 (30.0)
Mental rituals	10 (26.3)	13 (31.0)	16 (40.0)
Current medications			
Selective serotonin reuptake inhibitor	10 (26.3)	21 (50.0)	11 (27.5)
Antihistamine	1 (2.6)	2 (4.8)	2 (5.0)
Central stimulants	0	2 (4.8)	0
Antipsychotic	0	1 (2.4)	0
Sleep medication	0	1 (2.4)	0
Other antidepressant	0	0	1 (2.5)
Psychiatric comorbidities			
Depression	7 (18.4)	2 (4.8)	3 (7.5)
Social anxiety disorder	4 (10.5)	3 (7.1)	2 (5.0)
Attention-deficit/hyperactivity disorder	0	3 (7.1)	0
Panic disorder	2 (5.3)	1 (2.4)	2 (5.0)
Agoraphobia	1 (2.6)	1 (2.4)	3 (7.5)
Borderline personality disorder	1 (2.6)	0	0
Generalized anxiety disorder	1 (2.6)	8 (19.0)	7 (17.5)
Specific phobia	0	0	1 (2.5)
Health anxiety disorder	1 (2.6)	1 (2.4)	1 (2.5)
Tic disorder	1 (2.6)	0	1 (2.5)
Trichotillomania	0	1 (2.4)	3 (7.5)

Abbreviations: CBT, cognitive behavioral therapy; ICBT, internet-based cognitive behavioral therapy; OCD, obsessive-compulsive disorder.

### Primary Outcome

The estimated mean difference between therapist-guided ICBT and face-to-face CBT was 2.10 points (90% CI, −0.41 to 4.61 points) on the Y-BOCS, meaning that the mean Y-BOCS score for ICBT was 2.1 points higher, favoring face-to-face CBT, at the primary end point (3-month follow-up). This difference was not statistically significant (time-by-group interaction effect estimates, *Z* = 1.38; SE = 0.05; *d* = 0.54 [95% CI, −0.25 to 1.33]; *P* = .17); however, the 90% CI included both 0 (no estimated difference) and the prespecified noninferiority margin of 3 points, meaning that the primary noninferiority results were inconclusive.

**Figure 2.**
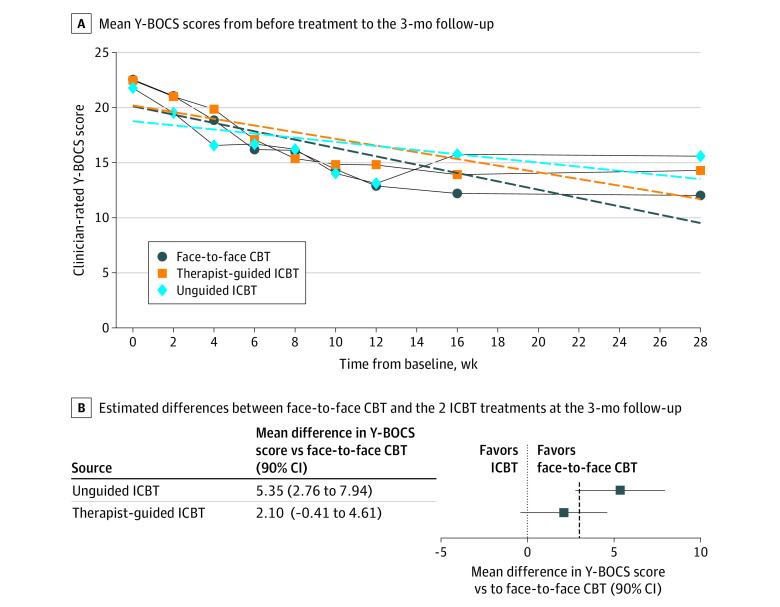
Yale-Brown Obsessive Compulsive Scale (Y-BOCS) Scores Over Time Panel A shows clinician-rated observed (solid lines) and estimated (dashed lines) Y-BOCS scores from before treatment to the 3-month follow-up. Panel B shows estimated differences between face-to-face cognitive behavioral therapy (CBT) and the 2 internet-based cognitive behavioral therapy (ICBT) treatments at the 3-month follow-up. The dotted line indicates the prespecified noninferiority margin of 3 points on the Y-BOCS.

For unguided ICBT, the estimated Y-BOCS difference compared with face-to-face CBT was 5.35 points (90% CI, 2.76-7.94 points), meaning that unguided ICBT was inferior to face-to-face CBT, and this difference was statistically significant (time-by-group interaction effect estimates, *Z* = 3.39; SE = 0.06; *d* = 1.38 [95% CI, 0.56-2.19]; *P* < .001). However, the 90% CIs overlapped the noninferiority threshold of 3 points (90% CI, 2.76-7.94 points), which made inference regarding the noninferiority margin inconclusive (Figure 2B). The observed and estimated Y-BOCS scores from baseline to the primary end point are shown in Figure 2A and eTable 5 in Supplement 2. All 3 treatment groups had statistically significant improvements from baseline to the primary end point with large effect sizes (eTable 5 and eAppendix 8 in Supplement 2). The Little test for missing data was calculated for Y-BOCS from pretreatment to 3-month follow-up and was not statistically significant (120 participants; χ^2^_213_ = 220.6; *P* = .35), supporting the assumption that data were missing completely at random. There were missing Y-BOCS data at the primary end point (18% in the face-to-face CBT group, 5% in therapist-guided ICBT group, and 12% in the unguided ICBT group).

There was no moderating effect of source of referral on overall Y-BOCS change over time (time-by-source of referral interaction effect, *Z* = 0.03 [95% CI, −0.06 to 0.13]; SE = 0.05; *P* = .53), or change over time across groups (therapist-guided ICBT, *Z* = −0.13 [95% CI, −0.35 to 0.09]; SE = 0.11; *P* = .26; unguided ICBT, *Z* = 0.04 [95% CI, −0.19 to 0.27]; SE = 0.12; *P* = .74). As sensitivity analyses, the main Y-BOCS model was fitted with a quadratic time effect (eAppendix 9 and eFigure 3 in Supplement 2), and the moderating effect of site was evaluated (eAppendix 10 in Supplement 2).

### Secondary Outcomes

There were significant interaction effects on the MADRS-S, the Global Assessment of Functioning, CGI-I, and Sheehan Disability Scale at the 3-month follow-up between therapist-guided ICBT and face-to-face CBT, favoring face-to-face CBT. For unguided ICBT compared with face-to-face CBT, there were significant interaction effects for all outcome measures, except the Y-BOCS–Self-Rated, at the 3-month follow-up, always favoring face-to-face CBT (Table 2 and eTable 6, eTable 7, eTable 8, and eTable 9 in Supplement 2).

**Table 2.** Change in Symptoms From Baseline to the 3-Month Follow-up for Secondary Outcomes

Outcome	Change from pretreatment, *B* (SE)^a^			Group contrasts, Cohen *d* (95% CI)^b^		
	Face-to-face CBT	Guided ICBT	Unguided ICBT	Face-to-face CBT vs guided ICBT	Face-to-face CBT vs unguided ICBT	Guided ICBT vs unguided ICBT
Obsessive-Compulsive Inventory–Revised						
Posttreatment	−11.55 (1.43)	−9.27 (1.32)	−7.47 (1.37)	0.21 (−0.53 to 0.94)	0.17 (−0.57 to 0.91)	−0.03 (−0.74 to 0.68)
3-mo follow-up	−12.73 (1.45)	−10.78 (1.34)	−7.54 (1.41)	0.15 (−0.59 to 0.89)	0.37 (−0.39 to 1.12)	0.22 (−0.51 to 0.94)
Yale-Brown Obsessive-Compulsive Scale–Self-Rated						
Posttreatment	−7.32 (0.98)	−7.43 (0.9)	−5.72 (0.95)	0.13 (−0.52 to 0.78)	0.29 (−0.37 to 0.96)	0.16 (−0.47 to 0.8)
3-mo follow-up	−8.51 (1)	−8.5 (0.92)	−5.98 (0.97)	0.16 (−0.5 to 0.82)	0.53 (−0.15 to 1.21)	0.37 (−0.28 to 1.02)
Montgomery-Åsberg Depression Rating Scale Self-Rated						
Posttreatment	−4.41 (1.1)	−2.19 (1)	−2.38 (1.05)	0.02 (−0.77 to 0.8)	−0.06 (−0.86 to 0.74)	−0.08 (−0.84 to 0.69)
3-mo follow-up	−6.05 (1.11)	−2.48 (1.02)	−2.16 (1.09)	0.33 (−0.47 to 1.12)	0.37 (−0.44 to 1.18)	0.04 (−0.74 to 0.82)
EuroQol 5-Dimensions						
Posttreatment	0.06 (0.04)	0.06 (0.03)	0.02 (0.03)	0.02 (−0.59 to 0.63)	0 (−0.63 to 0.62)	−0.03 (−0.62 to 0.57)
3-mo follow-up	0.12 (0.04)	0.06 (0.03)	0.02 (0.04)	−0.41 (−1.03 to 0.21)	−0.4 (−1.04 to 0.23)	0 (−0.61 to 0.62)
Global Assessment of Functioning						
Posttreatment	6.69 (1.52)	5.32 (1.37)	4.25 (1.43)	0.26 (−0.34 to 0.87)	−0.06 (−0.68 to 0.55)	−0.33 (−0.92 to 0.26)
3-mo follow-up	9.64 (1.53)	4.44 (1.37)	4.91 (1.44)	−0.35 (−0.96 to 0.26)	−0.44 (−1.07 to 0.19)	−0.09 (−0.68 to 0.51)
Sheehan Disability Scale						
Posttreatment	−5.43 (1.08)	−3.08 (0.96)	−2.87 (1)	0.2 (−0.54 to 0.93)	0.21 (−0.53 to 0.96)	0.02 (−0.7 to 0.73)
3-mo follow-up	−6.84 (1.09)	−3.23 (0.98)	−3.11 (1.03)	0.5 (−0.25 to 1.24)	0.5 (−0.27 to 1.26)	0 (−0.73 to 0.73)
Insomnia Severity Index, Post^c^	−0.95 (0.76)	−1.54 (0.68)	−0.84 (0.7)	−0.36 (−1.23 to 0.5)	−0.21 (−1.09 to 0.67)	0.15 (−0.69 to 0.99)

Abbreviations: CBT, cognitive behavioral therapy; ICBT, internet-based cognitive behavioral therapy.

^a^ Pretreatment to follow-up least squares means were based on mixed-effects models with a random intercept, fixed effects of time and group, and interaction effect time by group. The *B* coefficient is estimated using comparisons posttreatment minus pretreatment and follow-up minus pretreatment, respectively, within each group.

^b^ Between-group effect sizes were calculated using the least-squares means from mixed-effects models, using the residual SD of random effects as sigma. Comparisons are presented as B minus A so that positive values indicate a larger improvement for group A. Note that for EuroQol 5-dimensions and Global Assessment of Functioning, where an increase in total score indicates improvement, a negative effect size coefficient indicates larger improvements for group A.

^c^ Insomnia Severity Index was measured at pretreatment and posttreatment only; hence, there is no 3-month results for this outcome.

### Cost-effectiveness Analysis

**Figure 3.**
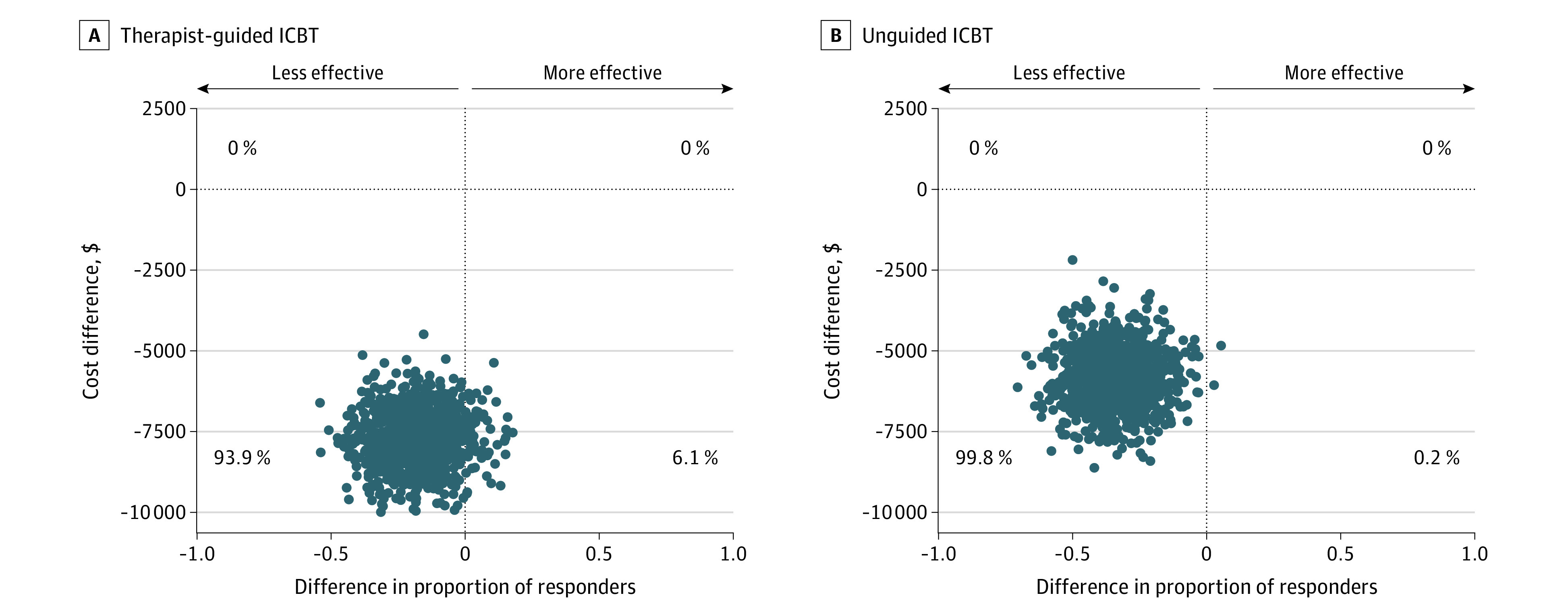
Cost-effectiveness Planes at the 3-Month Follow-up Graphs show before treatment to 3-month follow-up cost-effectiveness planes of therapist guided internet-based cognitive behavioral therapy (ICBT) (A) and unguided ICBT (B) compared with face-to-face CBT for obsessive-compulsive disorder. Costs are from a societal perspective and based on the Trimbos and Institute of Medical Technology Assessment Cost Questionnaire on Costs Associated with Psychiatric Illness using Swedish health care tariff listings. The effect is shown as the rate of response, defined as a 35% or more reduction in the Yale-Brown Obsessive Compulsive Scale from before treatment and a Clinical Global Impression–Improvement score of 1 or 2.

Therapists in the face-to-face CBT group spent a mean of 120.4 (95% CI, 115.4-125.3) minutes per week delivering treatment and 10.6 (95% CI, 8.9-12.4) minutes per week in the guided ICBT group. In the unguided ICBT group, a fixed time for administrative tasks was set to 60 minutes for the entire treatment period. Altogether, this led to an estimated mean (SE) treatment cost of $6795 ($237) for face-to-face CBT, compared with $603 ($176) for guided ICBT and $249 ($168) for unguided ICBT (details on costs are shown in eTable 10, eTable 11, eTable 12, eTable 13, eTable 14, and eTable 15 in Supplement 2). Both ICBT groups incurred substantial cost savings (range, $6190-$6593) per treated participant compared with face-to-face CBT. When expanding to a full societal perspective, the cost savings were estimated to be $6153 (95% CI, $4536-$7563; *P* < .001) for guided ICBT and $5761 (95% CI, $4145-$7298; *P* < .001) for unguided ICBT compared with face-to-face CBT. Figure 3 displays the cost-effectiveness and clinical effectiveness distribution of both guided and unguided ICBT vs face-to-face CBT (cost-effectiveness planes at posttreatment and 12-month follow-up are shown in eFigure 4, eFigure 5, eFigure 6, and eFigure 7 in Supplement 2, and cost-utility planes are shown in eFigure 8, eFigure 9, eFigure 10, eFigure 11, eFigure 12, and eFigure 13 in Supplement 2).

Fifteen nonresponders in the ICBT treatment groups (7 in the guided ICBT group and 8 in the unguided ICBT) received additional face-to-face CBT from the 3-month to the 12-month follow-up assessments. This led to a mean yearly cost of $1696 per participant in the guided IBCT group and $1172 in the unguided ICBT group, representing cost savings of 75% and 83%, respectively, compared with face-to-face CBT. Both ICBT groups incurred lower costs than the face-to-face CBT group but with similar clinical efficacy at the 12-month follow-up (eTable 16 and eAppendix 11 in Supplement 2).

### Post Hoc Analyses

At the primary end point, the proportion of participants classified as responders in the face-to-face CBT and guided ICBT groups was not significantly different (24 participants [77%] vs 18 participants [45%]; odds ratio [OR], 1.43 [95% CI, −0.88 to 3.74]; *P* = .31). By contrast, the proportion of responders was greater in the face-to-face group than in the unguided ICBT group (participants [77%] vs 5 participants [16%]; OR, 2.91 [95% CI, 0.33 to 5.49]; *P* = .02). The proportion of responders in the guided vs unguided ICBT groups did not differ significantly (18 participants [45%] vs 5 participants [16%]; OR, 1.48 [95% CI, −0.78 to 3.73]; *P* = .27). The proportion of participants in remission at the primary end point was higher in face-to-face CBT group (14 participants [45%]) compared with the guided ICBT group (6 participants [15%]) and unguided ICBT group (4 participants [10%]); ORs ranged from 0.42 to 2.0 (eAppendix 12 and eTable 17 in Supplement 2).

### Adverse Events

The most frequently reported adverse event during treatment was anxiety (30 participants [25%]), followed by depressive symptoms (20 participants [17%]), stress (11 participants [9%]), and sleep disturbances (9 participants [8%]) (eAppendix 13, eTable 18, and eTable 19 in Supplement 2). Two serious adverse events related to increased suicidal ideation (1 in the face-to-face treatment and 1 in the guided ICBT treatment condition) were recorded. Both participants were briefly admitted to the hospital as in-patients but remained in the trial.

## Discussion

To our knowledge, this RCT is the first direct comparison of both guided and unguided ICBT with individual face-to-face CBT for adults with OCD. Participants in all 3 treatment groups experienced significantly improved symptoms, and all groups also maintained their treatment gains up to the 12-month follow-up (nonresponders from both ICBT groups receiving additional face-to-face CBT included). The predefined noninferiority margin in this trial was 3 points on the Y-BOCS, a more conservative margin than previous noninferiority trials of OCD, which have used margins of 4 or 5 points.^10,33,34^ At the 3-month follow-up, the difference between therapist-guided ICBT and face-to-face CBT was 2.10 points on the Y-BOCS, favoring face-to-face CBT, and corresponding to a clinically marginal difference in symptom severity. However, the upper limit of the confidence intervals exceeded the noninferiority margin of 3, making the noninferiority evaluation inconclusive. Had we used a noninferiority margin of 5 points, we would have concluded that therapist-guided ICBT was noninferior to face-to-face CBT (eFigure 14 in Supplement 2). In contrast, unguided ICBT would have fallen short of the noninferiority margin.

At the primary end point, 77% of the participants in the face-to-face CBT and 45% in the guided ICBT group were classified as treatment responders, but only 16% in the unguided ICBT group were responders. Face-to-face CBT was significantly better than therapist-guided ICBT at improving the participants’ depressive symptoms and global functioning, but there were no significant interaction effects for secondary outcome measures at the primary end point. Unguided ICBT was significantly worse than face-to-face CBT with regard to all secondary outcome measures, except the Y-BOCS–Self-Rated, at the primary end point.

The costs of delivering therapist-guided ICBT ($603) and unguided ICBT ($249) were substantially lower than that for face-to-face CBT ($6795). The health economic evaluation indicated that both ICBT treatments were cost-effective compared with face-to-face CBT also when broadening the perspective to include all direct medical and societal costs, in line with a recent trial that investigated internet-delivered CBT for children and adolescents with OCD.^10^

### Strengths and Limitations

The trial had high participant retention and minimal data loss. Ratings by independent assessors showed that the face-to-face treatment was delivered in a highly competent way with effect sizes on par with those in previous trials.^37^ The same therapists treated participants across treatment conditions, minimizing risk of individual therapist factors confounding the treatment effects. The randomization sequence was generated by an independent unit, which also monitored the trial according to good clinical practice principles. Masking integrity checks showed that assessors were truly blind to group allocation. Source of referral did not moderate treatment outcome with CBT, suggesting that ICBT is effective also for clinic-referred patients.

This trial also has limitations. First, although unguided ICBT did not incorporate any therapist support, it is possible that repeated contact with trial assessors could have served as prompts to engage with treatment, although no such direct instructions were provided. Second, the trial was powered specifically to detect differences in Y-BOCS, and findings from secondary outcomes should be considered exploratory. Third, although the impact of missing data was minimized by having repeated assessments for each outcome and maximum likelihood estimation in the statistical models, there were missing Y-BOCS data at the primary end point (18% in the face-to-face CBT group, 5% in the therapist-guided ICBT group, and 12% in the unguided ICBT group), which should be considered when evaluating the results. Fourth, the cost estimations were based on a self-rated scale and adapted in a Swedish tax-funded universal health setting and might not be applicable to other countries and health care systems. Fifth, the current trial excluded participants with comorbidities such as autism and our results are not fully generalizable to all patients seen in regular psychiatric care.

## Conclusions

Although this RCT could not conclusively demonstrate noninferiority, the findings suggest that therapist-guided ICBT is a cost-effective alternative to face-to-face CBT for adults with OCD in scenarios where traditional CBT is not available. Unguided ICBT is probably less efficacious but could be an alternative when providing remote clinician support is not feasible.

## Notes

Supplement 1.
Trial Protocol and Statistical Analysis Plan

Supplement 2. **eTable 1. **Inclusion and Exclusion Criteria **eAppendix 1.** Adherence and Competence Ratings **eAppendix 2.** Description of Treatments **eFigure 1.** Online Treatment Platform, Text-Based Main View **eFigure 2.** Online Treatment Platform, Homework Report **eTable 2.** Description of Therapists **eTable 3.** Assessment Overview **eAppendix 3.** Psychometric Properties of Scales Used **eAppendix 4.** Statistical Analyses Continued **eAppendix 5.** Cost Categories **eAppendix 6.** Dropout and Missing Data **eTable 4.** Standardized Mean Differences in Baseline Participant Characteristics **eAppendix 7.** Statistically Significant Baseline Participants Characteristics as Covariates **eTable 5.** Within-Group Estimated Means and Effect Sizes of Y-BOCS **eAppendix 8.** Long-term Effects of Y-BOCS **eAppendix 9.** Y-BOCS Mixed Effects Model With Quadratic Time Effect **eFigure 3.** Y-BOCS Line Graph With Quadratic Time Effect **eAppendix 10.** Site as a Covariate in the Main Y-BOCS Model **eTable 6.** Observed Means and Standard Deviations **eTable 7.** Between-Group Effect Size Contrasts at Posttreatment **eTable 8.** Long-term Effects of Secondary Outcomes **eTable 9.** Long-term Effects of CGI-S and CGI-I **eTable 10.** Treatment Platform Usage **eTable 11.** Cost Tariffs **eTable 12.** Detailed Costs Breakdown at Pretreatment **eTable 13.** Detailed Costs Breakdown at Posttreatment **eTable 14.** Detailed Costs Breakdown at 3-Month Follow-up **eTable 15.** Detailed Costs Breakdown at 12-Month Follow-up **eFigure 4.** Cost-effectiveness of Therapist-Guided ICBT Versus Face-to-Face CBT at Posttreatment **eFigure 5.** Cost-effectiveness of Therapist-Guided ICBT Versus Face-to-Face CBT at 12-Month Follow-up **eFigure 6.** Cost-effectiveness of Unguided ICBT Versus Face-to-Face CBT at Posttreatment **eFigure 7.** Cost-effectiveness of Unguided ICBT Versus Face-to-Face CBT at 12-Month Follow-up **eFigure 8.** Cost-Utility of Therapist-Guided ICBT Versus Face-to-Face CBT at Posttreatment **eFigure 9.** Cost-Utility of Therapist-Guided ICBT Versus Face-to-Face CBT at 3-Month Follow-up **eFigure 10.** Cost-Utility of Therapist-Guided ICBT Versus Face-to-Face CBT at 12-Month Follow-up **eFigure 11.** Cost-Utility of Unguided ICBT Versus Face-to-Face CBT at Posttreatment **eFigure 12.** Cost-Utility of Unguided ICBT Versus Face-to-Face CBT at 3-Month Follow-up **eFigure 13.** Cost-Utility of Unguided ICBT Versus Face-to-Face CBT at 12-Month Follow-up **eTable 16.** Treatment Costs **eAppendix 11.** Costs of Crossover Treatment **eAppendix 12.** Response and Remission Rates **eTable 17.** Long-term Response and Remission Rates **eAppendix 13.** Adverse Events (AEs) **eTable 18.** Adverse Events During Study Period **eTable 19.** Adverse Events During Treatment **eFigure 14.** Y-BOCS Noninferiority Figure With Multiple Margins and CIs
eReferences

Supplement 3.
Data Sharing Statement
