# Error in Figure 2B

**DOI:** 10.1001/jamanetworkopen.2022.22742

**Published:** 2022-07-01

In the Original Investigation titled “Effect of Internet-Based vs Face-to-Face Cognitive Behavioral Therapy for Adults With Obsessive-Compulsive Disorder: A Randomized Clinical Trial,”^1^ published March 14, 2022, there was an error in Figure 2B, with numbers for unguided vs therapist-guided internet-based cognitive behavioral therapy transposed. This article has been corrected.^1^

## References

[1] Lundström L, Flygare O, Andersson E, . Effect of internet-based vs face-to-face cognitive behavioral therapy for adults with obsessive-compulsive disorder: a randomized clinical trial. JAMA Netw Open. 2022;5(3):e221967. doi:10.1001/jamanetworkopen.2022.1967 35285923 PMC9907343

