# Error in Results

**DOI:** 10.1001/jamanetworkopen.2023.23948

**Published:** 2023-06-30

In the Original Investigation titled “Effect of Internet-Based vs Face-to-Face Cognitive Behavioral Therapy for Adults With Obsessive-Compulsive Disorder: A Randomized Clinical Trial,”^1^ published March 14, 2022, incorrect numbers of participants were shown in the Post Hoc Analyses subsection of the Results; the incorrect numbers were removed. This article has been corrected.^1^
