# Supplementary material for: Effect of Internet-Based vs Face-to-Face Cognitive Behavioral Therapy for Adults With Obsessive-Compulsive Disorder: A Randomized Clinical Trial
Source: JAMA Netw Open. 2022 Mar 14;5(3):e221967. doi: 10.1001/jamanetworkopen.2022.1967 (PMC9907343; doi:10.1001/jamanetworkopen.2022.1967)
Supplement: Supplement 1. — Trial Protocol and Statistical Analysis Plan [file jamanetwopen-e221967-s001.pdf]

Study protocol for a single-blind, randomized controlled, non-inferiority trial of Internet-based versus face-to-face cognitive behaviour therapy for obsessive-compulsive disorder

Christian Rück[1,2], Lina Lundström[1,2], Oskar Flygare[1], Jesper Enander[1,2], Matteo Bottai[3], David Mataix-Cols[1,2], Erik Andersson[1]

[1] Centre for Psychiatry Research, Department of Clinical Neuroscience, Karolinska Institutet, SE-171 77 Stockholm, Sweden; [2] Stockholm Health Care Services, Stockholm County Council, SE-14186 Stockholm, Sweden; [3] Institute of Environmental Medicine, Biostatistics, Karolinska Institutet, SE-171 77 Stockholm, Sweden

Corresponding author:

Christian Rück
Karolinska Institutet
M46
SE-14186 Huddinge
E-mail: christian.ruck@ki.se
Telephone: +46704843392

ABSTRACT

Introduction Expert guidelines recommend cognitive behaviour therapy (CBT) as a first line treatment for Obsessive-Compulsive Disorder (OCD) but the majority of patients with OCD do not have access to CBT. Internet-delivered CBT (ICBT) has the potential to make this evidence-based treatment more accessible whilst requiring less therapist time than traditional face-to-face (f2f) CBT. Data from six clinical trials suggests that ICBT for OCD is both efficacious and cost effective but whether ICBT is non-inferior to traditional f2f CBT for OCD is yet unknown.

Methods and analysis A single-blind, randomized controlled non-inferiority trial comparing therapist-guided ICBT, unguided ICBT, and individual (f2f) CBT for adult OCD patients. The primary objective is to investigate whether ICBT is non-inferior to gold standard f2f CBT. Secondary objectives are to investigate if ICBT is equally effective when delivered unguided, to establish the cost-effectiveness of ICBT, and to investigate if the treatment outcome differs between self-referred and clinically-referred patients. Participants will be recruited at two specialist OCD clinics in Stockholm, and also through online self-referral. Participants will be randomized to one of three treatment conditions: F2f CBT, ICBT with therapist support or unguided ICBT. The total number of participants will be 120 and masked assessments will be administered at baseline, bi-weekly during treatment, at post-treatment, and at 3- and 12-months follow-ups. The main outcome measure is the clinician-rated Yale-Brown Obsessive Compulsive Scale (Y-BOCS) at 3-month follow-up. The margin of non-inferiority is set to 3 points on the Y-BOCS using a 90% confidence interval.

Ethics and dissemination The study has been approved by the Regional Ethics Board of Stockholm (REPN 2015/1099-31/2) and registered at Clinicaltrials.gov (NCT02541968). The study will be reported in accordance with the CONSORT statement for non-pharmacological trials. The results will be published in peer-reviewed academic journals and disseminated to patient organizations and media.

ARTICLE SUMMARY

- First study evaluating if two modalities of ICBT are non-inferior to the gold standard face-to-face CBT for OCD.
- Full health economic evaluation of therapist-guided ICBT, unguided ICBT and f2f CBT for OCD.
- Recruitment of both clinic-referred and self-referred patients, which will help generalise the results to more typical OCD cases.
- The exclusion of participants with certain diagnoses, e.g. people with Autism Spectrum Disorder, limits the generalizability.

INTRODUCTION

Obsessive-Compulsive Disorder (OCD) is a mental disorder characterized by obsessions (e.g., 'did I really lock that door?'), and compulsions (e.g., repeatedly checking that a door is locked). OCD affects ~2% of the general population(1) and is associated with poor quality of life, functional impairment across multiple life domains, high suicide risk,(2) and a large societal economic burden.(3) The disorder usually onsets before the age of 25 and has a low probability of remission if left untreated.(4)

Cognitive behavioural therapy (CBT) is currently recommended by the NICE guidelines as a first-line treatment for OCD.(5) Unfortunately, there is a gap between supply and demand of CBT for OCD; barriers to treatment access include a shortage of trained CBT therapists,(6) costs associated with treatment, geographical barriers, and embarrassment to openly disclose one's OCD symptoms.(7) Specialized CBT for OCD is therefore not accessible for most patients and only a minority of sufferers (5-10%) receive this evidence-based treatment.(8)

Internet cognitive behaviour therapy (ICBT) has the advantage of being more accessible and requiring less therapist time than face to face (f2f) CBT, potentially resulting in savings for the health care system. In therapist-guided ICBT, the patient logs on to a secure website and works with written self-help materials and homework assignments. During the treatment, the patient receives asynchronous online support by an identified therapist, who motivates the patient and troubleshoots any problems that may occur during the treatment. Therapist-guided ICBT has the potential to increase access to evidence based care and there is a substantial body of work demonstrating that therapist-guided ICBT can increase access to treatment for several mental disorders without impairing efficacy. In a recent meta-analysis where therapist-guided ICBT was compared to face-to-face CBT for both somatic and psychiatric disorders, therapist-guided ICBT was shown to have comparable efficacy to traditional f2f CBT treatment.(9) At the Internet psychiatry unit in Stockholm (www.internetpsykiatri.se), the effectiveness of therapist-guided ICBT for psychiatric disorders within clinical psychiatric care has been evaluated with positive long-term effects.(10-12)

Our research group has previously developed and tested therapist-guided ICBT for adults with OCD.(13-16) In a first pilot study of therapist-guided ICBT for OCD (n=23), large within-group effects (d = 1.56) were found for ICBT.(15) In a subsequent RCT (n=101), therapist-guided ICBT was superior to an attention control condition with a large between-group effect size (d = 1.12).(13) The treatment effects were sustained up to two years after treatment.(16) In a third study (n=128), therapist-guided ICBT for OCD, with or without the addition of the partial NMDA-agonist d-cycloserine was investigated. Although no significant effect of d-cycloserine was found, large within-group improvements were observed for both groups: d-cycloserine (d= 1.82) and placebo (d=2.20).(14) Therapist-guided ICBT for OCD has also shown positive results across cultures and age groups. In Australia, Wootton and colleagues and Mahoney and colleagues have both shown therapist-guided ICBT for OCD to be effective in randomized controlled trials.(17, 18) In Germany, Herbst and colleagues have tested therapist-guided ICBT for OCD with positive long-term effects.(19) ICBT is also efficacious and cost effective in adolescents with OCD (20, 21)

There is some evidence to suggest that ICBT can be delivered without any therapist involvement.(18, 22-24) However, this contradicts earlier literature suggesting that OCD patients receiving therapist support have lower attrition and fare better in treatment.(25) If ICBT could be entirely unguided, even more patients could receive help at a minimal cost.

Remaining evidence gaps that need to be closed

Although multiple research groups have found that therapist-guided ICBT is a promising approach for treating OCD, there are several critical issues that need to be addressed before the implementation of ICBT in a regular health care context can be recommended. **First**, it is unclear if ICBT is non-inferior to gold standard f2f CBT. **Second**, we do not know if our ICBT treatment is equally effective when delivered unguided. **Third**, there are no high-quality cost-effectiveness studies on ICBT for OCD and it is crucial to make a full health economical evaluation of ICBT vs. the gold standard f2f CBT. **Fourth**, the existing studies supporting the efficacy of ICBT in OCD have all relied on self-referred subjects. Self-referred subjects may be less complex, have better insight into their difficulties and be more motivated for treatment and therefore potentially affecting the generalizability of previous findings.(26) **Fifth**, since we do not yet know for whom ICBT is particularly suitable, the identification of reliable predictors and moderators of treatment outcome aid in choosing the right treatment from the start.

Aims and objectives

Primary objective

1. Our primary objective is to establish whether ICBT is non-inferior to f2f CBT with regard to OCD symptoms (measured with the masked clinician-rated Yale-Brown Obsessive Compulsive Scale, Y-BOCS).

Secondary objectives

2. To investigate if ICBT for OCD can be delivered without therapist support without impairing efficacy.
3. To determine if ICBT, compared to f2f CBT, is a cost-effective treatment for OCD.
4. To examine if there is a difference in treatment outcome between self-referred and clinically referred patients.
5. To explore predictors and moderators of treatment outcome as a first step towards personalized treatment selection.

METHODS AND ANALYSIS

Study design

Single-blind, randomized controlled non-inferiority trial comparing therapist-guided ICBT, unguided ICBT without therapist support, and individual f2f CBT for OCD in adults. The total number of participants will be 120 (40 per group), with stratification according to source of referral (self- vs. clinic referred patients). Block randomization will be performed within each stratum to ensure all participants are equally represented across treatment conditions. Participants will be assessed at baseline, bi-weekly during treatment, at post-treatment, and at 3- and 12-months follow-ups. The CONSORT flowchart of the trial is depicted in Figure 1.

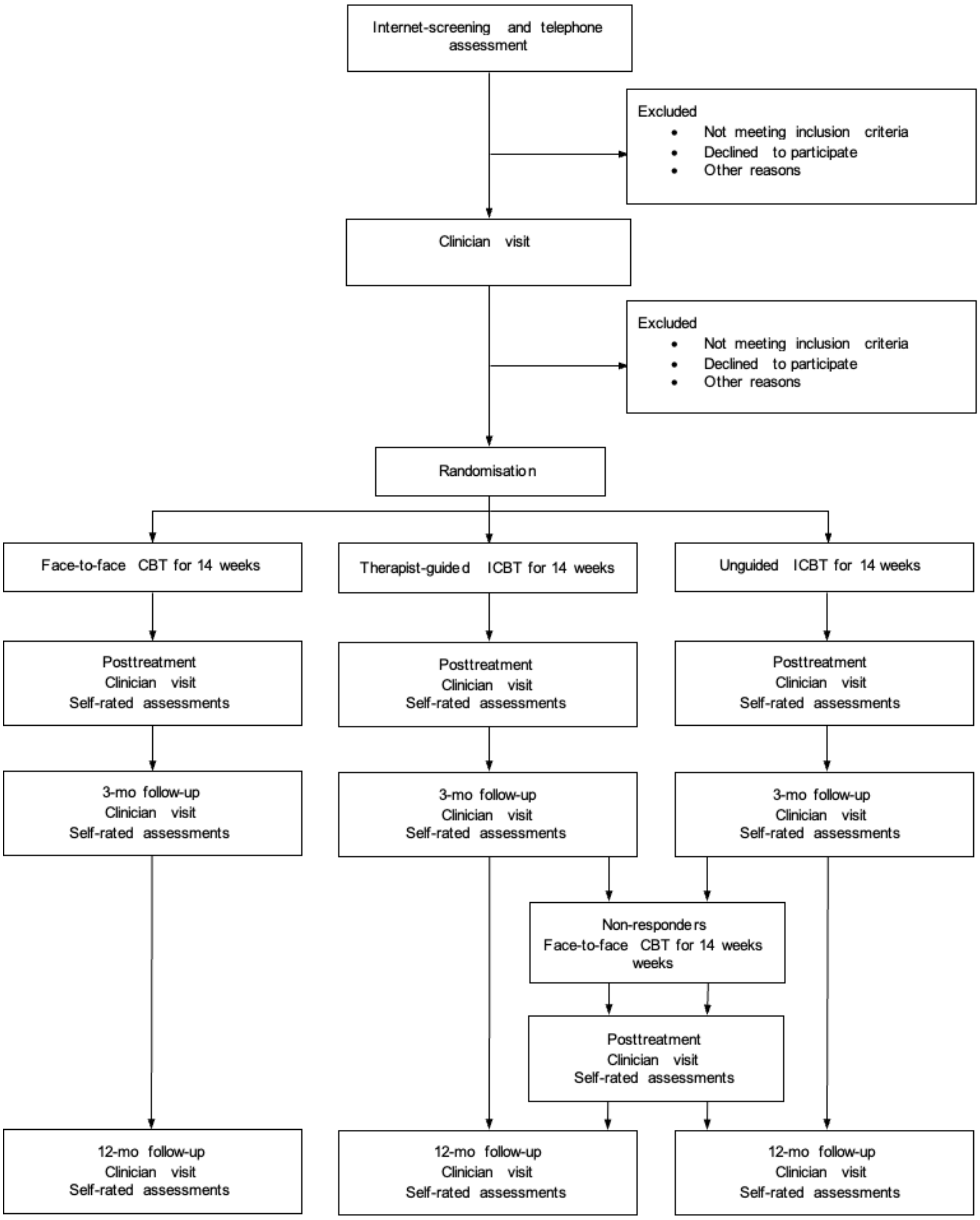

Figure 1: Participant flow in the study

Sample selection

Regular patients referred to two OCD specialist clinics in Stockholm will be assessed for eligibility. The trial will also be advertised online so that interested participants can self-refer by registering on the trial's secure webpage and completing a screening questionnaire. People living in Stockholm, Södermanland or Uppsala County are eligible to participate in the study (these counties are within 1 to 2 hours travel distance to Stockholm).

After completing an online screening, a clinical psychologist will contact potentially suitable participants by telephone for a brief screening interview. They will then be offered an appointment with a psychiatrist at one of the two OCD specialist clinic for a full psychiatric assessment. The psychiatrist will administer the Mini International Neuropsychiatric Interview (MINI)(27) and The Structured Clinical Interview for DSM-5 (SCID-5)(28) to

confirm the diagnosis of OCD, document psychiatric comorbidities, administer baseline instruments, and decide on inclusion/exclusion. Table 1 lists inclusion and exclusion criteria.

Table 1. Overview of inclusion and exclusion criteria

Inclusion criteria	≥ 18 years of age
	Primary diagnosis of OCD according to DSM-5
	Internet access
	Written consent of participation in the study
Exclusion criteria	Other psychological treatment for OCD during the treatment period
	Completed CBT for OCD in the last 12 months
	Changes in psychotropic medication within the last two months
	Bipolar disorder
	Psychosis
	Alcohol or substance dependence
	Autism spectrum disorder
	Organic brain disorder
	Hoarding disorder or OCD with primary hoarding symptoms
	Suicidal ideation
	Subjects that lack the ability to read written Swedish or lack the cognitive ability to assimilate the written material

Randomization and concealment

The randomization sequence will be generated by Karolinska Trial Alliance (KTA, https://karolinskatrialalliance.se, an independent entity not involved in the study) before inclusion of the first participant, using masked block randomization. Patients will receive their randomization number based on the order of their first psychiatrist appointment. Patients will be stratified based on self- or clinical referral. Sealed envelopes with information on treatment allocation will be stored in a secure locker in case of emergency unblinding.

Assessors will be blind to group assignment up to the 12- month follow-up. To ensure that the blinding is maintained, patients will be given clear instructions not to disclose which treatment they have been randomized to while being interviewed by the blind assessors. Where blindness is inadvertently broken, raters will be immediately replaced and the participant re-assessed by another rater. Blind raters will be asked to guess each patient's group allocation at each assessment point.(29) This will establish if the blind raters' guesses regarding treatment allocation were better than chance.

Interventions

Therapist-guided ICBT. Patients will receive 14 weeks of ICBT for OCD using a validated treatment protocol.(13-16) As in regular CBT for OCD, the main treatment component is exposure and response prevention (ERP). The therapists will be licensed psychologists with expertise in treating patients with OCD. Therapists will respond to messages encrypted in the Internet platform at a set time during office hours (8am-5pm) on weekdays, in order to ensure that participants receive a response within 24 hours. Each participant's response rate at the 3-month follow-up will be calculated and monitored by the project leaders. The participants who are non-responders (defined as Y-BOCS reduction < 35% and CGI-I >2)(30) at the 3-month follow-up will be contacted by telephone and offered face-to-face CBT for 14 weeks.

Self-guided ICBT. This arm will be identical to the ICBT described above but without any online therapist support. If participants experience any technical problems with the online platform during the treatment, they can contact project leaders for help. In the internet platform, patients will have detailed contact information in case of emergency. Participants in this group who are non-responders at 3-month follow-up will be offered up to 14 weeks of f2f CBT according to the same procedure explained in the previous section.

Individual f2f CBT. Patients receive 16 sessions of individual f2f CBT for OCD delivered over a time period of 14 weeks, according to a validated protocol.(31) Sessions will be held twice weekly during the first two weeks and once a week for the remaining 12 weeks. The therapists will be licensed psychologists with expertise in treating patients with OCD. The content of the f2f CBT is the same as in the ICBT arms. Sessions will be audiotaped in order to ensure that the therapists adhere to the treatment protocol. Adherence to protocol will be independently rated by a psychologist (not otherwise involved in the study) specialized in CBT treatment for OCD.

Sample size calculation

In order to provide accurate estimates for the power calculation in the current trial, we will used individual-level data from a previous study of therapist-guided ICBT with identical Y-BOCS assessments by blinded raters and six repeated observations.(14) To calculate the required sample size, we used a bootstrap simulation with 1000 samples using the following assumptions, based on data from the previous trial: a variance of the random intercept of 10.5, a variance for the random slope of 0.04, and a within- individual residual variance of 20.4. With 3 treatment groups and 8 observations (Y-BOCS) per patient, we estimated that a total of 120 participants would be needed to detect a slope difference between two groups (i.e. group 1 vs. group 2 and group 1 vs. group 3) of 3 points at 3-month follow-up with over 90% power. We will request an interim power analysis by the Karolinska Trial Alliance to test these assumptions, using data from 80 individuals, and adjust sample size if power is lower than anticipated (see supplementary file 1 for a detailed description).

Measurements

Table 2 lists clinician-rated and self-rated assessments at the different time points. The primary outcome measure is the clinician-rated Yale-Brown Obsessive Compulsive Scale (Y-BOCS), the gold standard for assessing the severity of OCD symptoms.(32) Clinicians in this trial will practice together on case examples to establish high inter-rater reliability. The Y-BOCS will be administered by blind raters at baseline, at weeks 2, 4, 6, 8, 10 and 12 during treatment, at post-treatment (week 15), and at 3- and 12-months follow-ups. The primary endpoint is the 3-month follow-up.

Secondary clinician-administered outcome measures are the Clinical Global Impression – Severity and Improvement scale (CGI-S, CGI-I),(33) the Structured Clinical Interview for DSM-5 (SCID-5), obsessive–compulsive and related disorders,(28) and the Global Assessment of Functioning (GAF).(34) Secondary self-rated outcome measures are the Obsessive-Compulsive Inventory (OCI-R),(35) the self-rated Y-BOCS,(32) the Montgomery-Åsberg Depression Rating Scale (MADRS-S),(36) Sheehan Disability Scale (SDS)(37) and the Euroqol (EQ-5D).(38) The Patient Exposure/Responsprevention Adherence Scale (PEAS)(39) will be used to quantify compliance with ERP homework and the Working Alliance Inventory – Short Form (WAI-SF)(40) will be used to measure therapeutic alliance in the face-to-face CBT and ICBT with therapist support treatment conditions. The Insomnia Severity Index (ISI)(41) will be used to measure participants sleep patterns and the Treatment

Credibility Scale (TCS)(42) will be used to measure how credible participants perceive the treatment to be. Measurements will be administered before and after treatment as well as during 3- and 12 months follow-ups. In order to increase participant retention at follow-up assessments, participants will be notified via text message 48 hours prior to an appointment. Should a participant not attend a follow-up session, a psychiatrist will contact participants via telephone to perform the assessments.

Table 2. Assessments at different time points

	Screening	Pre treatment	During treatment	Post treatment	3 month follow-up	12 month follow-up
**Clinician-rated instruments**						
SCID-5 (OCD)	X	X		X	X	X
Y-BOCS	X	X	X	X	X	X
CGI-S		X		X	X	X
CGI-I				X	X	X
GAF		X		X	X	X
SMURF			X	X	X	X
PEAS			X	X		
MADRS-S			X			
MINI		X				
**Self-rated instruments**						
Y-BOCS	X	X		X	X	X
Y-BOCS checklist	X					
OCI-R	X	X		X	X	X
EQ-5D	X	X		X	X	X
EQ-5D index	X	X		X	X	X
Audit	X					
Dudit	X					
MADRS-S	X	X		X	X	X
PHQ9	X					
SDS	X	X		X	X	X
ASRS	X					
ISI		X		X		
TiC-P		X		X	X	X
TCS			X			
WAI-SF			X	X		

* SCID-5, The Structured Clinical Interview for DSM 5; Y-BOCS, Yale-Brown Obsessive Compulsive Scale; CGI-S, Clinical Global Impression-Severity scale; CGI-I, Clinical Global Impression-Improvement scale; GAF, Global Assessment of Functioning; SMURF, Safety Monitoring Uniform Report Form; PEAS, Patient Exposure/Responsprevention Adherence Scale; MADRS-S Montgomery-Åsberg Depression Rating Scale; MINI, Mini International Neuropsychiatric Interview; OCI-R, Obsessive-Compulsive Inventory-Revised; EQ-5D, EuroQol 5 Dimension scale; AUDIT, Alcohol Use Disorder Identification Test; DUDIT, Drug Use Disorders Identification Test; PHQ9, Patient Health Questionnaire; SDS, Sheehan Disability Scale; ASRS, Adult ADHD Self Report Scale; ISI, Insomnia Severity Index; TIC-P, Treatment Inventory of Costs in Psychiatric Patients; TCS, Treatment Credibility Scale; WAI-SF, Working Alliance Inventory – Short Form.

Safety and adverse events

Data on adverse events and suicidal ideation will be collected by blinded independent raters bi-weekly during treatment, at post-treatment and at 3- and 12-month follow-up. Adverse

events will be collected using a standardized checklist, the Safety Monitoring Uniform Report Form (SMURF).(43) If a participant expresses suicidal ideation (i.e. a score on item 9 of the MADRS-S ≥ 4), assessors will initiate a structured suicide risk assessment. If there is an urgent need for psychiatric care, a trial psychiatrist will contact participants to schedule a face-to-face appointment as soon as possible.

Statistical analysis

The main outcome analyses will be conducted according to the "intent-to-treat" principle. Mixed-effects regression analyses for repeated measures with maximum likelihood estimation (MLE) of parameters will be used with the assumption that data are missing at random. The latter assumption will be tested. For each outcome measure, the model will include fixed effects of time (baseline, mid-treatment, post-treatment, and 3-month follow-up [primary endpoint]), treatment group (guided ICBT, unguided ICBT, f2f CBT) and an interaction effect of treatment group x time to allow for the differential change between the three groups from baseline to the 3-month follow-up. The models will include individuals' random intercept and random slope to account for variability between and within participants over time. Within- and between-group effect sizes will be calculated with Cohen's *d*.(44) Numbers needed to treat will be calculated based on responder status.

Alpha for all analyses will be set at 0.05. Non-inferiority is established when the 90% Wald confidence interval for the difference between treatment conditions excludes the pre-specified margin of inferiority, which is set at 3 points on the Y-BOCS (45, 46). This means that if the upper limit of the 90% confidence interval is less than 3 points, we are 95% confident that ICBT will be non-inferior to f2f CBT. The non-inferiority hypothesis will be tested of both therapist-guided and self-guided ICBT against the f2f CBT. Additional analyses of the 12-month follow-up data will determine whether the treatment gains are maintained long-term and whether ICBT is non-inferior to f2f CBT at follow-up.

Cost-effectiveness analysis

Health economic data will be collected using the TIC-P(47) and the Swedish National Patient Register, the Swedish Prescribed Drugs Register and the longitudinal integrated data-base for health insurance and work-related research (LISA). Costs will be analysed using a societal perspective i.e. including both sick-leave, hospitalizations, service use, medication, etc. and analysed in relation to outcome (i.e. OCD symptoms and quality-adjusted life years using the Y-BOCS and EQ-5D, respectively). National tariffs will be used to estimate costs from health care visits. Productivity losses will be estimated using gross earnings data from each patient.(48) Treatment costs, i.e. therapist support time per patient logged on the platform and time spent on f2f sessions, will be included in the cost estimation.

Cost-effectiveness comparisons will be analysed using incremental cost-effectiveness ratios. The "net benefit approach" will also be used. This approach estimates the cost-effectiveness depending on different societal willingness-to-pay values for one unit of improvement.(48) Non-parametric bootstrapping (one thousand replications) will be used to estimate the difference between ICBT (guided or unguided) and gold standard f2f CBT.

Analysis of predictors and moderators

We will analyse predictors and moderators of response and remission status at 3- and 12-month follow-up using repeated k-fold cross validation with 10 folds and 20 repeats to reduce the risk of model instability (49, 50). We then average model performance over the repeats using area under the receiver operating characteristic curve (AUC) of sensitivity and specificity to

distinguish between responders/remitters and non-responders/non-remitters (51).

Limitations

There are several potential threats to the validity and generalizability of the current trial results, some of which apply to most clinical trials. First, the trial was designed to maximise the chances of the results being as generalizable as possible. However, despite the best of our efforts to recruit both clinic- and self-referred individuals, it will be difficult to confidently claim that our participants will be representative of the entire population of OCD patients in Sweden. For example, we will not know if our results are generalizable to patients with comorbid autism spectrum disorder or to patients who are too ill to seek help and participate in clinical studies. Second, it is impossible to conduct double-blinded clinical trials of behavioural interventions. In an effort to increase transparency, our design includes careful checks of the extent to which raters are blind to the group allocation. Third, while our study is well powered to test the non-inferiority hypothesis, it may not be powered to test the same hypothesis for all secondary measures or for the cost-effectiveness calculations. Fourth, patients in the unguided ICBT arm still have contact with health care professionals at baseline, during treatment and after treatment (e.g., bi-weekly telephone assessments, post-treatment and follow-up appointments). An entirely unguided treatment would involve limited or no contact with health care professionals.

Patient and public involvement

We received input from patients from three previous OCD internet CBT trials which guided the design of the current study. In the current trial, no patients were involved in the design of the study or in the decision of outcome measures. Neither will patients be involved in the recruitment of participants or in the decision of the research question. A patient organization for OCD and related disorders (the Swedish OCD Foundation) will be involved in the recruitment of participants by informing their members about the study. We will assess the burden of the trial interventions on the patients by collecting information about adverse events, quality of life, and time spent on the treatment. We will gather information about the patients satisfaction with treatment through an online self-rating questionnaire at the end of treatment. We plan to disseminate the results of the research to study participants and to the Swedish OCD Foundation.

ETHICS AND DISSEMINATION

The trial will be conducted in compliance with this study protocol, the Declaration of Helsinki and Good Clinical Practice (GCP). Karolinska Trial Alliance (KTA) is an external party that will monitor the study every 6 months and ensure that the study follows GCP, i.e. that all participants give informed written consent and that study related materials are handled correctly. All professionals involved in the study will attend a course in GCP and get certified by the KTA.

The study has been approved by the Regional Ethics Board of Stockholm (REPN 2015/1099-31/2) and registered at Clinicaltrials.gov (NCT02541968), and will be reported in accordance with the CONSORT statement for non-pharmacological trials.(52) Ethical risks are deemed minimal and both f2f CBT and ICBT have well-documented efficacy.

Current trial status

Recruitment of participants started in September 2015 and the last participant is expected to reach the primary end-point (3-month follow-up) in February 2019. Primary data analysis will begin in April 2019. The naturalistic follow-up phase of the trial will continue until November 2019.

CONCLUSION

OCD is associated with significant suffering, loss of function across multiple life domains, high suicide risk, and large societal costs. ICBT has great potential to increase access to evidence-based care for a large group of sufferers that normally do not receive evidence-based psychological treatments. The study outlined in this protocol is the first direct comparison of ICBT and gold standard f2f CBT and is a crucial step before ICBT can be recommended for use within the regular health-care system. The study will provide new insights into the effectiveness of different treatment modalities for OCD and the health economic evaluation will help decision-makers to rationally allocate available resources. Implementation of ICBT in regular healthcare would dramatically increase the availability of effective treatment to those suffering from OCD.

Contributors

CR and LL wrote the first draft of the paper. All authors: CR, LL, OF, DMC, MB, EA and JE conceived the study and revised the manuscript for relevant scientific content. MB specifically revised the statistical analyses and power calculation sections of the paper. All authors approved the final version of the manuscript.

Competing interests

All authors declare: no support from any organization for the submitted work; no financial relationships with any organizations that might have an interest in the submitted work in the previous three years; no other relationships or activities that could appear to have influenced the submitted work.

Funding
This study is funded by the Swedish Research Council (K2013-61P-22168) and the regional agreement on medical training and clinical research (ALF) between Stockholm County Council and Karolinska Institutet.

FIGURE CAPTIONS

Figure 1. CONSORT-flow diagram

REFERENCES

1. Fullana MA, Vilagut G, Rojas-Farreras S, Mataix-Cols D, de Graaf R, Demyttenaere K, et al. Obsessive–compulsive symptom dimensions in the general population: Results from an epidemiological study in six European countries. Journal of Affective Disorders. 2010;124(3):291-9.

2. Fernández de la Cruz L, Rydell M, Runeson B, D'Onofrio BM, Brander G, Rück C, et al. Suicide in obsessive–compulsive disorder: a population-based study of 36 788 Swedish patients. Molecular Psychiatry. 2016;22:1626.

3. Huppert JD, Simpson HB, Nissenson KJ, Liebowitz MR, Foa EB. Quality of life and functional impairment in obsessive–compulsive disorder: a comparison of patients with and without comorbidity, patients in remission, and healthy controls. Depression and Anxiety. 2009;26(1):39-45.

4. Rasmussen SA, Eisen JL. Epidemiology of obsessive compulsive disorder. J Clin Psychiatry. 1990;51 Suppl:10-3; discussion 4.

5. Obsessive N. Compulsive Disorder: core interventions in the treatment of obsessive–compulsive disorder and body dysmorphic disorder. London: NICE. 2005.

6. Larsson BPM, Kaldo V, Broberg AG. Similarities and differences between practitioners of psychotherapy in Sweden: A comparison of attitudes between psychodynamic, cognitive, cognitive–behavioral, and integrative therapists. Journal of Psychotherapy Integration. 2009;19(1):34-66.

7. Shapiro DA, Cavanagh K, Lomas H. GEOGRAPHIC INEQUITY IN THE AVAILABILITY OF COGNITIVE BEHAVIOURAL THERAPY IN ENG LAND AND WALES. Behavioural and Cognitive Psychotherapy. 2003;31(2):185-92.

8. Goodwin R, Koenen KC, Hellman F, Guardino M, Struening E. Helpseeking and access to mental health treatment for obsessive-compulsive disorder. Acta Psychiatr Scand. 2002;106(2):143-9.

9. Andersson G, Cuijpers P, Carlbring P, Riper H, Hedman E. Guided Internet-based vs. face-to-face cognitive behavior therapy for psychiatric and somatic disorders: a systematic review and meta-analysis. World Psychiatry. 2014;13(3):288-95.

10. El Alaoui S, Hedman E, Kaldo V, Hesser H, Kraepelien M, Andersson E, et al. Effectiveness of Internet-based cognitive–behavior therapy for social anxiety disorder in clinical psychiatry. Journal of consulting and clinical psychology. 2015;83(5):902.

11. Hedman E, Ljótsson B, Kaldo V, Hesser H, El Alaoui S, Kraepelien M, et al. Effectiveness of Internet-based cognitive behaviour therapy for depression in routine psychiatric care. Journal of Affective Disorders. 2014;155:49-58.

12. Hedman E, Ljótsson B, Rück C, Bergström J, Andersson G, Kaldo V, et al. Effectiveness of Internet-based cognitive behaviour therapy for panic disorder in routine psychiatric care. Acta Psychiatrica Scandinavica. 2013;128(6):457-67.

13. Andersson E, Enander J, Andren P, Hedman E, Ljotsson B, Hursti T, et al. Internet-based cognitive behaviour therapy for obsessive-compulsive disorder: a randomized controlled trial. Psychol Med. 2012;42(10):2193-203.

14. Andersson E, Hedman E, Enander J, Radu Djurfeldt D, Ljotsson B, Cervenka S, et al. D-Cycloserine vs Placebo as Adjunct to Cognitive Behavioral Therapy for Obsessive-Compulsive Disorder and Interaction With Antidepressants: A Randomized Clinical Trial. JAMA Psychiatry. 2015;72(7):659-67.

15. Andersson E, Ljotsson B, Hedman E, Kaldo V, Paxling B, Andersson G, et al. Internet-based cognitive behavior therapy for obsessive compulsive disorder: a pilot study. BMC Psychiatry. 2011;11:125.

16. Andersson E, Steneby S, Karlsson K, Ljotsson B, Hedman E, Enander J, et al. Long-term efficacy of Internet-based cognitive behavior therapy for obsessive-compulsive disorder with or without booster: a randomized controlled trial. Psychol Med. 2014;44(13):2877-87.

17. Wootton BM, Dear BF, Johnston L, Terides MD, Titov N. Remote treatment of obsessive-compulsive disorder: A randomized controlled trial. Journal of Obsessive-Compulsive and Related Disorders. 2013;2(4):375-84.

18. Mahoney AE, Mackenzie A, Williams AD, Smith J, Andrews G. Internet cognitive behavioural treatment for obsessive compulsive disorder: A randomised controlled trial. Behav Res Ther. 2014;63C:99-106.

19. Herbst N, Voderholzer U, Thiel N, Schaub R, Knaevelsrud C, Stracke S, et al. No talking, just writing! Efficacy of an Internet-based cognitive behavioral therapy with exposure and response prevention in obsessive compulsive disorder. Psychotherapy and psychosomatics. 2014;83(3):165-75.

20. Lenhard F, Ssegonja R, Andersson E, Feldman I, Ruck C, Mataix-Cols D, et al. Cost-effectiveness of therapist-guided internet-delivered cognitive behaviour therapy for paediatric obsessive-compulsive disorder: results from a randomised controlled trial. BMJ Open. 2017;7(5):e015246.

21. Lenhard F, Andersson E, Mataix-Cols D, Rück C, Vigerland S, Högström J, et al. Therapist-Guided, Internet-Delivered Cognitive-Behavioral Therapy for Adolescents With Obsessive-Compulsive Disorder: A Randomized Controlled Trial. Journal of the American Academy of Child & Adolescent Psychiatry.56(1):10-9.e2.

22. Hauschildt M, Schröder J, Moritz S. Randomized-controlled trial on a novel (meta-)cognitive self-help approach for obsessive-compulsive disorder (“myMCT”). Journal of Obsessive-Compulsive and Related Disorders. 2016;10:26-34.

23. Wootton BM, Dear BF, Johnston L, Terides MD, Titov N. Self-guided internet administered treatment for obsessive-compulsive disorder: Results from two open trials. Journal of Obsessive-Compulsive and Related Disorders. 2014;3(2):102-8.

24. Wootton BM, Dear BF, Johnston L, Terides MD, Titov N. Self-guided internet-delivered cognitive behavior therapy (iCBT) for obsessive–compulsive disorder: 12 month follow-up. Internet Interventions. 2015;2(3):243-7.

25. Kenwright M, Marks I, Graham C, Franses A, Mataix-Cols D. Brief scheduled phone support from a clinician to enhance computer-aided self-help for obsessive-compulsive disorder: randomized controlled trial. J Clin Psychol. 2005;61(12):1499-508.

26. Mataix-Cols D, Cameron R, Gega L, Kenwright M, Marks IM. Effect of referral source on outcome with cognitive-behavior therapy self-help. Compr Psychiatry. 2006;47(4):241-5.

27. Lecrubier Y, Sheehan DV, Weiller E, Amorim P, Bonora I, Harnett Sheehan K, et al. The Mini International Neuropsychiatric Interview (MINI). A short diagnostic structured interview: reliability and validity according to the CIDI. European Psychiatry. 1997;12(5):224-31.

28. First MB. Structured Clinical Interview for the DSM (SCID). The Encyclopedia of Clinical Psychology: John Wiley & Sons, Inc.; 2014.

29. Basoglu M, Marks I, Livanou M, Swinson R. Double-blindness procedures, rater blindness, and ratings of outcome. Observations from a controlled trial. Arch Gen Psychiatry. 1997;54(8):744-8.

30. Mataix-Cols D, Fernandez de la Cruz L, Nordsletten AE, Lenhard F, Isomura K, Simpson HB. Towards an international expert consensus for defining treatment response, remission, recovery and relapse in obsessive-compulsive disorder. World psychiatry : official journal of the World Psychiatric Association (WPA). 2016;15(1):80-1.

31. Yadin E, Foa, E. Lichner, T. . Exposure and Response (Ritual) Prevention for Obsessive-Compulsive Disorder: Therapist Guide: Oxford University Press; 2012.

32. Goodman WK, Price LH, Rasmussen SA, Mazure C, Fleischmann RL, Hill CL, et al. The Yale-Brown Obsessive Compulsive Scale. I. Development, use, and reliability. Arch Gen Psychiatry. 1989;46(11):1006-11.

33. Guy W. Clinical global impression scale. The ECDEU Assessment Manual for Psychopharmacology-Revised Volume DHEW Publ No ADM. 1976;76(338):218-22.

34. Spitzer RL, Gibbon M, Williams J, Endicott J. Global assessment of functioning (GAF) scale. Outcome assessment in clinical practice. 1996:76-8.

35. Foa EB, Huppert JD, Leiberg S, Langner R, Kichic R, Hajcak G, et al. The Obsessive-Compulsive Inventory: Development and validation of a short version. Psychological Assessment. 2002;14(4):485-96.

36. Svanborg P, Asberg M. A comparison between the Beck Depression Inventory (BDI) and the self-rating version of the Montgomery Asberg Depression Rating Scale (MADRS). J Affect Disord. 2001;64(2-3):203-16.

37. Sheehan D. Sheehan disability scale. Handbook of psychiatric measures. 2000:113-5.

38. EuroQol G. EuroQol--a new facility for the measurement of health-related quality of life. Health Policy. 1990;16(3):199-208.

39. Simpson HB, Maher M, Page JR, Gibbons CJ, Franklin ME, Foa EB. Development of a Patient Adherence Scale for Exposure and Response Prevention Therapy. Behavior therapy. 2010;41(1):30-7.

40. Tracey TJK, A. M. . Factor structure of the Working Alliance Inventory. Psychological Assessment: A Journal of Consulting and Clinical Psychology. 1989;1:207–10.

41. Bastien CH, Vallières A, Morin CM. Validation of the Insomnia Severity Index as an outcome measure for insomnia research. Sleep Medicine.2(4):297-307.

42. Devilly GJ, Borkovec TD. Psychometric properties of the credibility/expectancy questionnaire. Journal of Behavior Therapy and Experimental Psychiatry. 2000;31(2):73-86.

43. Greenhill LL, Vitiello B, Fisher P, Levine J, Davies M, Abikoff H, et al. Comparison of Increasingly Detailed Elicitation Methods for the Assessment of Adverse Events in Pediatric Psychopharmacology. Journal of the American Academy of Child & Adolescent Psychiatry. 2004;43(12):1488-96.

44. Cohen J. Statistical power analysis for the behavioural sciences. Hillside. NJ: Lawrence Earlbaum Associates. 1988.

45. Lovell K, Cox D, Haddock G, Jones C, Raines D, Garvey R, et al. Telephone administered cognitive behaviour therapy for treatment of obsessive compulsive disorder: randomised controlled non-inferiority trial. BMJ. 2006;333(7574):883.

46. Turner CM, Mataix-Cols D, Lovell K, Krebs G, Lang K, Byford S, et al. Telephone cognitive-behavioral therapy for adolescents with obsessive-compulsive disorder: a randomized controlled non-inferiority trial. J Am Acad Child Adolesc Psychiatry. 2014;53(12):1298-307 e2.

47. Hakkaart-van Roijen L, Van Straten A, Donker M, Tiemens B. Trimbos/iMTA questionnaire for costs associated with psychiatric illness (TIC-P). Rotterdam: Institute for Medical Technology Assessment. 2002.

48. Drummond MF, Sculpher MJ, Claxton K, Stoddart GL, Torrance GW. Methods for the economic evaluation of health care programmes: Oxford university press; 2015.

49. Molinaro AM, Simon R, Pfeiffer RM. Prediction error estimation: a comparison of resampling methods. Bioinformatics. 2005;21(15):3301-7.

50. Kim J-H. Estimating classification error rate: Repeated cross-validation, repeated hold-out and bootstrap. Computational Statistics & Data Analysis. 2009;53(11):3735-45.

51. Bradley AP. The use of the area under the ROC curve in the evaluation of machine learning algorithms. Pattern Recognition. 1997;30(7):1145-59.

52. Schulz KF, Altman DG, Moher D. CONSORT 2010 Statement: updated guidelines for reporting parallel group randomised trials. BMC Medicine. 2010;8(1):18.

STATISTIK
AKADEMIN

Anders Berglund, PhD 11 jan 2018, Uppsala

Interim power analysis to KTA

(Source interim-power-analysis-to-kta.pdf)

Real data

What to do:

1. Remove the hashes (#) in the code block below to make the code run as intended.
2. Select the attached CSV-file called **ICBTvsF2F_powercalc_data.csv**.
3. Run the code.
4. Report corresponding numbers from the output: variance of id, variance of time, residual variance.

```
# df_realdata <- read.csv(file.choose())
# fit_real <- lmer(total ~ (1 | id) + (1 | time), data = df_realdata) # summary(fit_real)
```

Results

- id has a variance of **12.77** (Random intercept)
- time has a variance of **10.02** (Random slope)
- The residual variance is **14.10** (Within-individual residual variance)

Statistikakademin AB Klostergatan 13 info@statistikakademin.se www.statistikakademin.se
753 21 Uppsala 018-410 82 82
