# Supplementary material for: Effect of Internet-Based vs Face-to-Face Cognitive Behavioral Therapy for Adults With Obsessive-Compulsive Disorder: A Randomized Clinical Trial
Source: JAMA Netw Open. 2022 Mar 14;5(3):e221967. doi: 10.1001/jamanetworkopen.2022.1967 (PMC9907343; doi:10.1001/jamanetworkopen.2022.1967)
Supplement: Supplement 2. — eTable 1. Inclusion and Exclusion Criteria eAppendix 1. Adherence and Competence Ratings eAppendix 2. Description of Treatments eFigure 1. Online Treatment Platform, Text-Based Main View eFigure 2. Online Treatment Platform, Homework Report eTable 2. Description of Therapists eTable 3. Assessment Overview eAppendix 3. Psychometric Properties of Scales Used eAppendix 4. Statistical Analyses Continued eAppendix 5. Cost Categories eAppendix 6. Dropout and Missing Data eTable 4. Standardized Mean Differences in Baseline Participant Characteristics eAppendix 7. Statistically Significant Baseline Participants Characteristics as Covariates eTable 5. Within-Group Estimated Means and Effect Sizes of Y-BOCS eAppendix 8. Long-term Effects of Y-BOCS eAppendix 9. Y-BOCS Mixed Effects Model With Quadratic Time Effect eFigure 3. Y-BOCS Line Graph With Quadratic Time Effect eAppendix 10. Site as a Covariate in the Main Y-BOCS Model eTable 6. Observed Means and Standard Deviations eTable 7. Between-Group Effect Size Contrasts at Posttreatment eTable 8. Long-term Effects of Secondary Outcomes eTable 9. Long-term Effects of CGI-S and CGI-I eTable 10. Treatment Platform Usage eTable 11. Cost Tariffs eTable 12. Detailed Costs Breakdown at Pretreatment eTable 13. Detailed Costs Breakdown at Posttreatment eTable 14. Detailed Costs Breakdown at 3-Month Follow-up eTable 15. Detailed Costs Breakdown at 12-Month Follow-up eFigure 4. Cost-effectiveness of Therapist-Guided ICBT Versus Face-to-Face CBT at Posttreatment eFigure 5. Cost-effectiveness of Therapist-Guided ICBT Versus Face-to-Face CBT at 12-Month Follow-up eFigure 6. Cost-effectiveness of Unguided ICBT Versus Face-to-Face CBT at Posttreatment eFigure 7. Cost-effectiveness of Unguided ICBT Versus Face-to-Face CBT at 12-Month Follow-up eFigure 8. Cost-Utility of Therapist-Guided ICBT Versus Face-to-Face CBT at Posttreatment eFigure 9. Cost-Utility of Therapist-Guided ICBT Versus Face-to-Face CBT at 3-Month Follow-up eFigure 10. Cost-Ut [file jamanetwopen-e221967-s002.pdf]

Supplemental Online Content

Lundström L, Flygare O, Andersson E, et al. Effect of internet-based vs face-to-face cognitive behavioral therapy for adults with obsessive-compulsive disorder: a randomized clinical trial. *JAMA Netw Open*. 2022;5(3):e221967. doi:10.1001/jamanetworkopen.2022.1967

eTable 1. Inclusion and Exclusion Criteria
eAppendix 1. Adherence and Competence Ratings
eAppendix 2. Description of Treatments
eFigure 1. Online Treatment Platform, Text-Based Main View
eFigure 2. Online Treatment Platform, Homework Report
eTable 2. Description of Therapists
eTable 3. Assessment Overview
eAppendix 3. Psychometric Properties of Scales Used
eAppendix 4. Statistical Analyses Continued
eAppendix 5. Cost Categories
eAppendix 6. Dropout and Missing Data
eTable 4. Standardized Mean Differences in Baseline Participant Characteristics
eAppendix 7. Statistically Significant Baseline Participants Characteristics as Covariates
eTable 5. Within-Group Estimated Means and Effect Sizes of Y-BOCS
eAppendix 8. Long-term Effects of Y-BOCS
eAppendix 9. Y-BOCS Mixed Effects Model With Quadratic Time Effect
eFigure 3. Y-BOCS Line Graph With Quadratic Time Effect
eAppendix 10. Site as a Covariate in the Main Y-BOCS Model
eTable 6. Observed Means and Standard Deviations
eTable 7. Between-Group Effect Size Contrasts at Posttreatment
eTable 8. Long-term Effects of Secondary Outcomes
eTable 9. Long-term Effects of CGI-S and CGI-I
eTable 10. Treatment Platform Usage
eTable 11. Cost Tariffs
eTable 12. Detailed Costs Breakdown at Pretreatment
eTable 13. Detailed Costs Breakdown at Posttreatment
eTable 14. Detailed Costs Breakdown at 3-Month Follow-up
eTable 15. Detailed Costs Breakdown at 12-Month Follow-up
eFigure 4. Cost-effectiveness of Therapist-Guided ICBT Versus Face-to-Face CBT at Posttreatment
eFigure 5. Cost-effectiveness of Therapist-Guided ICBT Versus Face-to-Face CBT at 12-Month Follow-up
eFigure 6. Cost-effectiveness of Unguided ICBT Versus Face-to-Face CBT at Posttreatment
eFigure 7. Cost-effectiveness of Unguided ICBT Versus Face-to-Face CBT at 12-Month Follow-up
eFigure 8. Cost-Utility of Therapist-Guided ICBT Versus Face-to-Face CBT at Posttreatment
eFigure 9. Cost-Utility of Therapist-Guided ICBT Versus Face-to-Face CBT at 3-Month Follow-up
eFigure 10. Cost-Utility of Therapist-Guided ICBT Versus Face-to-Face CBT at 12-Month Follow-up
eFigure 11. Cost-Utility of Unguided ICBT Versus Face-to-Face CBT at Posttreatment
eFigure 12. Cost-Utility of Unguided ICBT Versus Face-to-Face CBT at 3-Month Follow-up
eFigure 13. Cost-Utility of Unguided ICBT Versus Face-to-Face CBT at 12-Month Follow-up
eTable 16. Treatment Costs
eAppendix 11. Costs of Crossover Treatment
eAppendix 12. Response and Remission Rates
eTable 17. Long-term Response and Remission Rates
eAppendix 13. Adverse Events (AEs)
eTable 18. Adverse Events During Study Period
eTable 19. Adverse Events During Treatment
eFigure 14. Y-BOCS Noninferiority Figure With Multiple Margins and CIs
eReferences

This supplemental material has been provided by the authors to give readers additional information about their work.

© 2022 Lundström L et al. *JAMA Network Open.*

eTable 1. Inclusion and Exclusion Criteria

Inclusion criteria	≥ 18 years of age
	Primary diagnosis of OCD according to DSM-5
	Internet access
	Written consent of participation in the study
Exclusion criteria	Other psychological treatment for OCD during the treatment period
	Completed CBT for OCD in the last 12 months
	Changes in psychotropic medication within the last two months
	Bipolar disorder
	Psychosis
	Alcohol or substance dependence
	Autism spectrum disorder
	Organic brain disorder
	Hoarding disorder or OCD with primary hoarding symptoms
	Suicidal ideation
	Subjects that lack the ability to read written Swedish or lack the cognitive ability to assimilate the written material

eAppendix 1. Adherence and Competence Ratings

The interrater reliability for 24 sessions rated by both assessors The Cognitive Therapy Adherence and Competence Scale (CTACS)[35] was moderate (ICC = 0.49 [95% CI 0.19 – 0.71], p = .006). Adherence to treatment protocol was assessed by the same raters, who indicated whether techniques prescribed in the treatment manual occurred or not (for example homework review, exposure with response prevention).

A percentage of prescribed techniques used was then calculated for each session, and the average adherence was 92% [95% CI 88 – 96]. The interrater reliability for adherence was excellent (ICC = 0.90 [95% CI 0.81 – 0.95], $p < .001$).

eAppendix 2. Description of Treatments

Detailed Description of Face-to-Face Cognitive Behavior Therapy

The face-to-face CBT treatment was delivered according to a standard protocol of cognitive-behaviour therapy for OCD, based primary on exposure with response prevention (ERP).[29] The participants came to the clinic for 90-minute sessions twice a week during the first two weeks of treatment and one 90-minute session per week for the remaining 12 weeks, a total of 16 sessions. If a participant cancelled or did not show up for an appointment, the therapist was instructed to schedule a new appointment (i.e., more than one session per week) to compensate for the missed session. The treatment content was equivalent to that of ICBT, but mostly conveyed orally by the therapist, or by written worksheets. The participant was encouraged to work on homework exercises during the week, and homework assignments were reviewed by the therapist at the start of the next session.

The first four sessions included psychoeducation about OCD, worksheets to register obsessions and compulsions in the participants everyday life, goalsetting and psychoeducation about ERP. The remaining sessions involved ERP, where the therapist and participant conducted different ERP exercises at the clinic, outside the clinic or in the participants own home. The last session included a relapse prevention program with a summary of what the participant had learnt and a plan for how ERP practice would continue.

Detailed description of therapist- guided Internet-Delivered Cognitive Behavior Therapy (ICBT)

The content of the therapist-guided ICBT treatment was equivalent to that of regular face-to-face CBT for OCD . The treatment consisted of 10 modules with text chapters (figure 1) and associated work sheets with CBT exercises (figure 2). Module 1-4 contained psychoeducation about OCD with case examples of patients with different categories of obsessions and compulsions, worksheets to register obsessions and compulsions in the participants everyday life, common misinterpretations about obsessions, goalsetting and psychoeducation about exposure with response prevention (ERP). From module 5 and onwards, the participants were also instructed to perform everyday ERP exercises in their own home environment. Modules 6-9 covered ERP themes i.e., imaginal ERP and common pitfalls when conducting ERP and how to solve them. The last module consisted of a

© 2022 Lundström L et al. *JAMA Network Open.*

relapse prevention program with a summary of what the participants had learnt and a plan for how they could continue to practice ERP.[30]

Therapists answered questions and helped the participants to troubleshoot any problems that occurred throughout the treatment and supported the participants in doing their own ERP exercises. The correspondence between therapist and participant was through an email-like system within the internet platform. After finishing each module, the participants completed homework assignments by answering questions about the text and filling out work sheets with CBT exercises. Modules were unlocked and completed in consecutive order and the therapists gave written feed-back to the homework assignments before unlocking the next module.

Participants accessed the treatment platform through two-factor authentication using a password as well as a one-time code that was sent to the participant via SMS. Participants were free to log in to the platform at any time during the treatment period of 14 weeks, but were informed that they could expect a reply from a therapist within 24 hours on weekdays. Therapists communicated with the participants in the platform through an email-like message system and answered messages during office hours between 8:00-17:00. If a participant was inactive for more than 2-3 days, therapists sent a message to the participant in the platform. If no reply was given, an SMS was sent to encourage the participant to log in to the platform or ask for help. The therapist could also contact the participant via telephone if no response was given to prior contact attempts. To increase participant engagement in follow-up assessments, participants were reminded via text message 48 hours prior to an appointment. If a participant did not attend a follow-up session, a psychiatrist contacted the participant via telephone and performed the assessments. The participants were also reminded about the self-rated outcome measures via text messages sent from the internet platform.

Detailed Description of unguided Internet-Delivered Cognitive Behavior Therapy (ICBT)

The unguided ICBT treatment was delivered on the same platform and identical to the therapist-guided ICBT treatment, except there was no therapist involvement. Thus, it contained the same 10 modules, homework exercises, and worksheets described above. Participants were instructed to work with the modules in consecutive order, but all modules were unlocked at the start of treatment. Participants had to plan and conduct ERP exercises on their own, and they could answer homework assignments and send them in, but without getting a response back. No prompts to log in or engage with treatment were given to the participants during the treatment, even if they were inactive.

eFigure 1. Online Treatment Platform, Text-Based Main View

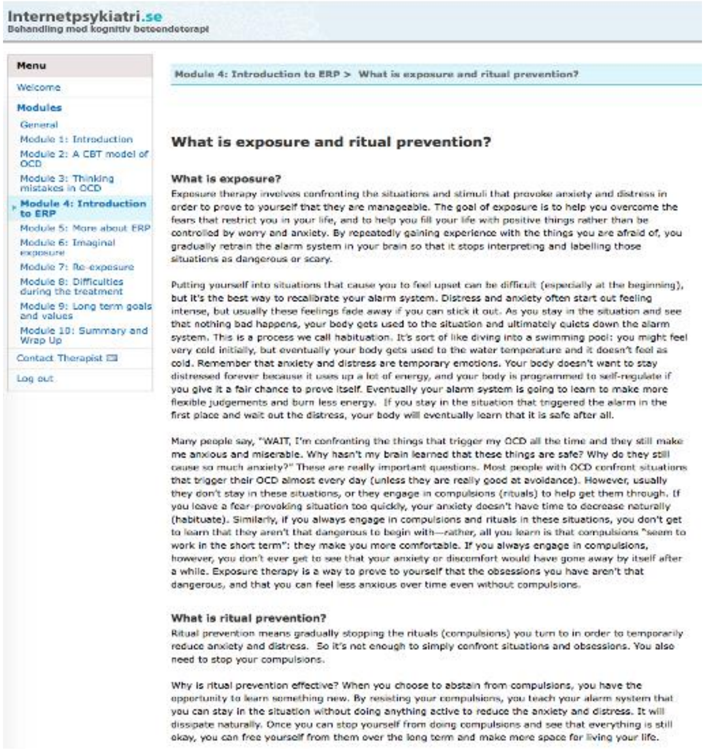

© 2022 Lundström L et al. *JAMA Network Open.*

eFigure 2. Online Treatment Platform, Homework Report

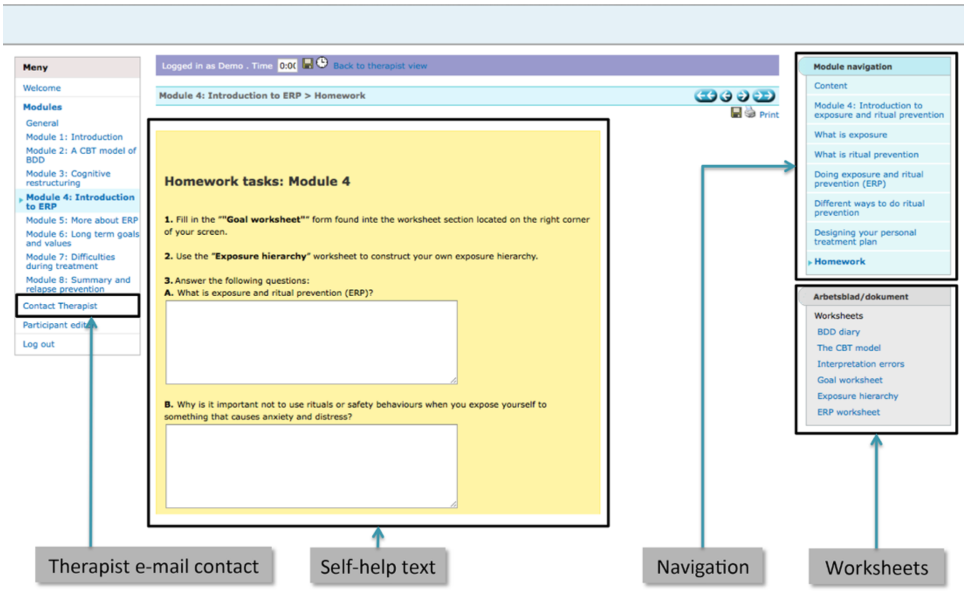

eTable 2. Description of Therapists

		Treated participants in group		Previous CBT experience		Year lic. Psychologist
Therapist	Profession	F2F - CBT	ICBT	F2F-CBT	ICBT	
A	Psychologist	yes	yes	yes	yes	2017
B	Psychologist	yes	yes	yes	yes	2013
C	Psychologist	yes	yes	yes	yes	2018
D	Psychologist	yes	yes	yes	yes	2015
E	Psychologist	yes	yes	yes	yes	2013
F	Psychologist	yes	no	yes	no	2015
G	Psychologist	yes	no	yes	no	2013
H	Psychologist	yes	yes	yes	yes	2017

eTable 3. Assessment Overview

	Screening	Pre treatment	During treatment	Post treatment	3 month follow-up	12 month follow-up
Clinician-rated instruments						
SCID-5 (OCD)	X	X		X	X	X
Y-BOCS	X	X	X	X	X	X
CGI-S		X		X	X	X
CGI-I				X	X	X
GAF		X		X	X	X
SMURF			X	X	X	X
PEAS			X	X		
MADRS-S			X			
MINI		X				
Self-rated instruments						

© 2022 Lundström L et al. *JAMA Network Open.*

Y-BOCS	X	X		X	X	X
Y-BOCS checklist	X					
OCI-R	X	X		X	X	X
EQ-5D	X	X		X	X	X
EQ-5D index	X	X		X	X	X
Audit	X					
Dudit	X					
MADRS-S	X	X		X	X	X
PHQ9	X					
SDS	X	X		X	X	X
ASRS	X					
ISI		X		X		
TiC-P		X		X	X	X
TCS			X			
WAI-SF			X	X		

SCID-5, The Structured Clinical Interview for DSM 5;[1] Y-BOCS, Yale-Brown Obsessive Compulsive Scale;[2] CGI-S, Clinical Global Impression-Severity scale; CGI-I, Clinical Global Impression-Improvement scale;[3] GAF, Global Assessment of Functioning;[4] SMURF, Safety Monitoring Uniform Report Form;[5] PEAS, Patient Exposure/Responsprevention Adherence Scale;[6] MADRS-S Montgomery-Åsberg Depression Rating Scale;[7] MINI, Mini International Neuropsychiatric Interview;[8] OCI-R, Obsessive-Compulsive Inventory-Revised;[9] EQ-5D, EuroQol 5 Dimension scale;[10] AUDIT, Alcohol Use Disorder Identification Test;[11] DUDIT, Drug Use Disorders Identification Test;[12] PHQ9, Patient Health Questionnaire;[13] SDS, Sheehan Disability Scale;[14] ASRS, Adult ADHD Self Report Scale; ISI, Insomnia Severity Index;[15] TIC-P, Treatment Inventory of Costs in Psychiatric Patients;[16] TCS, Treatment Credibility Scale;[17] WAI-SF, Working Alliance Inventory – Short Form.[18]

eAppendix 3. Psychometric Properties of Scales Used

The Yale Brown Obsessive Compulsive Scale (Y-BOCS) and the Yale Brown Obsessive Compulsive Scale - Self Report (Y-BOCS-SR)
The Yale Brown Obsessive Compulsive Scale is a self-reported version of the Y-BOCS and it comprises 10 items, rated on a 5-point Likert scale ranging from 0 (no symptoms) to 4 (severe symptoms). The total score ranges from 0 to 40. Y-BOCS has excellent inter-rater reliability and moderate to good internal consistency. There are moderate correlations between the clinician and the self-rating version of the Y-BOCS, where the obsession subscale has lower convergence between the two versions.[2,19]

The Global Assessment of Functioning (GAF)
The Global Assessment of Functioning (GAF) is a clinician rated measure of how much a person's symptoms affect their day-to-day life on a scale ranging from 0 to 100, where 100 represents superior functioning. The GAF have proved to be a reliable and valid measure of psychiatric disturbance in samples of severely mentally ill.[20]

Obsessive Compulsive Inventory - Revised (OCI-R)
The Obsessive Compulsive Inventory - Revised (OCI-R) is an 18 item self-report measure of OCD severity. It measures six different symptom dimensions of OCD and each item is scored on a scale from 0-4, with a total score of 72, with a higher score indicating more severe OCD. The OCI–R has excellent psychometric properties, and the subscales differentiate well between individuals with and without OCD.[9,21,22]

Montgomery Åsberg Depression Rating Scale - Self report (MADRS-S)
The Montgomery Åsberg Depression Rating Scale-Self report **(**MADRS-S) is a self-report measure of depression severity, with a total score ranging from 0 to 54, where a higher score indicates more severe depression. The scale consists of nine items, each measuring a different symptom on a seven-point scale. The MADRS-S has good to excellent test–retest reliability (r=0.80–0.94), as well as a high correlation (r=0.87) with the Beck Depression Inventory in a comparative study.[7,23]

© 2022 Lundström L et al. *JAMA Network Open.*

The euroqol (EQ-5D)
The euroqol (EQ-5D) is a self-reported generic measurement of global functioning and quality of life. It measures five health domains of importance to quality of life and each domain is scored from 0-3 where 0 indicates no problems and 3 indicates extreme problems. EQ-5D has good test-retest reliability (intraclass coefficient = .82 - .83) and acceptable convergent validity.[24]

The Clinical Global Impression (CGI)
The Clinical Global Impression (CGI) is a clinician-rated measure of clinical global severity of illness (CGI-S) and clinical global improvement (CGI-I). The CGI-S scores range from 1 (not at all ill) to 7 (extremely ill), and the CGI-I scores range from 1 (very much improved) to 7 (very much worse). CGI has shown good reliability and validity for a range of psychiatric disorders.[25,26]

The Insomnia Severity Index (ISI)
The Insomnia Severity Index (ISI), is a self-report instrument measuring a person's perception of his or her insomnia. The ISI comprises seven items and each item is scored on a 0–4 scale with a total score ranging from 0 to 28. A higher score indicates more severe insomnia. The ISI has adequate internal consistency and is a reliable self-report measure to evaluate perceived sleep difficulties.[15]

The Sheehan Disability Scale (SDS)
The Sheehan Disability Scale (SDS) is a brief instrument used to measure functional disability. The scale assesses functional impairment and disabilities in three domains and each domain is rated on a 0-10 scale where 10 indicates extreme impairment/disability. The Swedish SDS had a Cronbach's alpha coefficient of 0.77. Self-assessed SDS-scores correlated with expert rated GAF-scores (r = -0.606).[27]

The Credibility Expectancy Questionnaire
The Credibility Expectancy Questionnaire measures treatment credibility and expectancy for improvement. It is a self-report six item questionnaire where Items 1 to 3 and 5 are answered on a scale ranging from 1 (not at all) to 9 (very much) and items 4 and 6 are answered on a 0 (not at all) to 100% (very much) range. The questionnaire demonstrates high internal consistency within each factor and good test-retest reliability.[17]

The Working Alliance Inventory-Short Revised (WAI-SR)
The Working Alliance Inventory-Short Revised (WAI-SR) is a measure of therapeutic alliance, assessing three key aspects of the therapeutic alliance. The WAI-SR demonstrates good psychometric properties, with good reliability (alpha > 0.80) and good convergent validity (r > 0.64) with the Helping Alliance Questionnaire.[28]

eAppendix 4. Statistical Analyses Continued

Sample size calculation
The following assumptions was used: a variance of the random intercept of 10.5, a variance for the random slope of 0.04, and a within-individual residual variance of 20.4.[31]
We also requested an interim power analysis by an independent statistician at *Karolinska Trial Alliance* to test whether these estimates (using data from the first 80 participants) were accurate. Importantly, the interim analysis did not include the grouping variable, and only the variance estimates were reported. We concluded that the intercept, slope and variance were in line with our original power calculation (see supplementary file 2 for a detailed description).

Statistical analysis, continued
Effect sizes for Y-BOCS slope differences were derived from the main mixed-effects model, using the weekly slope difference between groups as the estimate, residual standard deviation as sigma, and residual degrees of freedom as the equivalent degrees of freedom to account for uncertainty in sigma.

The group x time interaction effect from the mixed-effects models of secondary outcomes were used to estimate the means and standardized effect sizes, using a superiority test to evaluate whether between-group comparisons differed from zero. Additional analyses included uncontrolled 12-month follow-up assessments in the models for secondary outcomes to determine whether the treatment gains were maintained in the long term.

For response and remission rates, group differences in the odds of response/remission were evaluated using Tukey's post hoc test of group differences with p-value adjustment for 3 groups. The numbers needed to treat were estimated based on the between-group differences in the proportions of responders/remitters, with confidence intervals based on the pooled standard error of the two groups being compared.

© 2022 Lundström L et al. *JAMA Network Open.*

For CGI-S and CGI-I, which were analyzed using ordinal logistic regression, mean class estimates were obtained for the values 1-7 based on the probability distribution of each outcome. By using ordinal logistic regression, we assume that the CGI ratings are discrete cut points from latent continuous variables measuring severity/improvement.

eAppendix 5. Cost Categories

Health economic data were collected and analyzed from health organizational, direct medical and societal perspectives. The health care organizational perspective covered the direct costs from therapist time spent per participant and costs for time spent on administration, travel and telephone calls by the therapists. For the unguided ICBT treatment, an estimated time of 60 minutes per participant, covering administrative tasks such as establishing the participant on the platform and sending reminders about assessments, was set for the entire treatment period. The second perspective included direct medical costs (medication usage and health care visits) and finally, the cost estimation was broadened to a full societal perspective including costs for unemployment, sick leave, and reductions in work and household abilities.

Participants reported costs for the past month in TIC-P,[16] which were then extrapolated to a 3-month period to cover the entire period between the pre-treatment, post-treatment, and 3-month assessments. Costs were analysed in relation to responder status based on the masked assessor-rated Y-BOCS[34] and quality-adjusted life years (QALYs) based on the EQ-5D.[10]

Tariffs from official listings for the publicly funded Swedish health care system were used to estimate the costs for health care visits, and costs for medications were calculated from market prices. Productivity losses (unemployment, sick leave, and reductions in work and household abilities) were estimated using the mean gross earnings,[36] and costs for domestic work reduction were derived from the domestic loss hourly tariff of €14.[37] All costs were computed in Swedish crowns and converted to US Dollars, based on power purchasing parities for the year 2017[38] when 8.55 Swedish crowns equalled $1.

The direct medical and societal costs that were based on the Treatment Inventory of Costs in Psychiatric Patients (TIC-P) questionnaire was carefully reviewed by two team members (LL and EA) without inclusion of the grouping variable or other patient identifiers, before being included in the cost estimation.

eAppendix 6. Dropout and Missing Data

Five participants in the face-to-face CBT group and one participant in the unguided ICBT treatment group were classified as study dropouts (defined as participants who did not complete any assessments from week six on). Removing those six participants did not alter the conclusions of the non-inferiority evaluation (Therapist-guided estimate = 1.98 [90% CI –0.53 to 4.50], Unguided estimate = 5.26 [90% CI 2.66 to 7.86].

Participants who completed treatment but did not provide data were categorized as missing at follow-up, and a total of 14 participants did not provide data on the Y-BOCS at 3-month follow-up (primary endpoint). As reported in the main manuscript, Little's test for missing data was not statistically significant, supporting the assumption that data were missing completely at random.

To further evaluate the mechanism of missingness, participants with (n = 106) and without (n = 14) Y-BOCS data at the 3-month follow-up were compared on all baseline participant characteristics presented in table 1. Participants with missing data did not differ on any demographic variables, medication status, or characteristics of their OCD. However, participants with missing data were more likely (4/14, 28.6%) than participants that provided Y-BOCS data (8/106, 7.5%) to have a co-morbid diagnosis of depression at baseline (*p* = .047). [32]

eTable 4. Standardized Mean Differences in Baseline Participant Characteristics

Standardized mean differences were estimated using the *ExtractSmd* function in the R-package *tableone* version 0.12.0. As described in the package documentation, several extensions are used for multivariate and multinomial variables and then summarized as weighted estimates.

For continuous variables, p-values for the F-value in ANOVA with group as an independent variable are shown. Chi-square test of independence was used to test group differences in categorical variables. P-values not adjusted for multiple testing. Statistically significant differences between groups were used to inform sensitivity analyses.

Variable	Face-to-face CBT vs Therapist-guided ICBT	Face-to-face CBT vs Unguided ICBT	Therapist-guided ICBT vs Unguided ICBT	Test of group difference, *p*
Source of referral	0,03	0,04	0,01	*p* = .983
Age	0,13	0,15	0,03	*p* = .781

Gender	0,03	0,09	0,12	*p* = .852
Age of onset	0,2	0,02	0,23	*p* = .524
Any previous treatment for OCD	0,18	0,31	0,13	*p* = .386
Previous suicide attempts	0,11	0,01	0,11	*p* = .871
Source of income	0,56	0,18	0,46	*p* = .35
Level of education	0,47	0,33	0,53	*p* = .493
Main obsessions and compulsions				
Aggressive	0,24	0,18	0,06	*p* = .542
Contamination	0,06	0,11	0,05	*p* = .891
Unacceptable thoughts	0,11	0,09	0,02	*p* = .867
Symmetry	0,11	0,15	0,04	*p* = .783
Washing	0,07	0,11	0,04	*p* = .884
Checking	0,2	0,08	0,12	*p* = .655
Ordering	0,29	0,09	0,2	*p* = .430
Mental rituals	0,1	0,29	0,19	*p* = .420
Current medications				
SSRI	0,5	0,03	0,47	*p* = .041
Antihistamine	0,11	0,12	0,01	*p* = .847
Central stimulants	0,32	0	0,32	*p* = .151
Anti-psychotic	0,22	0	0,22	*p* = .392
Sleep medication	0,22	0	0,22	*p* = .392
Other antidepressant	0	0,23	0,23	*p* = .365
Psychiatric comorbidities				
Depression	0,44	0,33	0,11	*p* = .103
Social anxiety disorder	0,12	0,21	0,09	*p* = .647
ADHD	0,39	0	0,39	*p* = .057
Panic disorder	0,15	0,01	0,14	*p* = .771
Agoraphobia	0,02	0,22	0,24	*p* = .433
Borderline personality disorder	0,23	0,23	0	*p* = .337
Generalized anxiety disorder	0,55	0,51	0,04	*p* = .062
Specific phobia	0	0,23	0,23	*p* = .365
Health anxiety disorder	0,02	0,01	0,01	*p* = .997
Tic disorder	0,23	0,01	0,23	*p* = .578
Trichotillomania	0,22	0,4	0,24	*p* = .167
Abbreviations: ADHD, Attention-deficit hyperactivity disorder; OCD, Obsessive-compulsive disorder; SSRI, Selective serotonin reuptake inhibitor.				

eAppendix 7. Statistically Significant Baseline Participants Characteristics as Covariates

The difference in proportion of participants prescribed SSRIs was statistically significant between the groups, with more participants in the therapist-guided ICBT group taking SSRIs compared to the two other groups. To investigate whether this affected the results, medication with SSRIs was included as a covariate in the linear mixed-effects model of Y-BOCS change over time from baseline to 3-month follow-up.

There was no significant moderation effect of SSRI medication on change over time for all groups combined (time x SSRI interaction effect Z = -0.08 [95% CI –0.25 to 0.09], SE = 0.09, *p* = 0.34), or for the therapist-guided ICBT group (time x group x SSRI interaction effect Z = 0.06 [95% CI –0.16 to 0.28], SE = 0.11, *p* = 0.58) and unguided ICBT group (time x group x SSRI interaction effect Z = -0.10 [95% CI –0.33 to 0.14], SE = 0.12, *p* = 0.42) when analyzed separately.

© 2022 Lundström L et al. *JAMA Network Open.*

eTable 5. Within-Group Estimated Means and Effect Sizes of Y-BOCS

	Change from pretreatment[1], *B* (SE)			Within-group effect size[2]. Cohen's *d* [95% CI]		
	Face-to-face CBT	Guided ICBT	Unguided ICBT	Face-to-face CBT	Guided ICBT	Unguided ICBT
Post	-6.06 (0.66)	-4.86 (0.58)	-3.01 (0.63)	-1.56 [-1.21 to -1.91]	-1.25 [-0.95 to -1.56]	-0.77 [-0.45 to -1.10]
3-month follow-up	-10.61 (1.16)	-8.51 (1.02)	-5.26 (1.10)	-2.73 [-2.13 to -3.33]	-2.19 [-1.66 to -2.72]	-1.35 [-0.79 to -1.92]

Explanations:

1- Estimated mean difference within each group compared to pre-treatment, using the main Y-BOCS model with fixed effects of time and group, their interaction, as well as random intercept and slope.

2- Within-group effect sizes calculated using the estimated means from the same mixed-effects model, using the residual standard deviation of random effects as sigma.

eAppendix 8. Long-term Effects of Y-BOCS

To evaluate the long-term effects on Y-BOCS, a mixed-effects model was fitted using fixed effects of group and time, their interaction, and a random intercept. Note that this model did not evaluate slope differences as the extended time between measurements at follow-up made such a modelling approach inappropriate. The estimates shown include cross-over treatments in both ICBT groups.

Gains were maintained at the 12-month follow-up for the face-to-face CBT group (B = -1.13, SE = 0.93, p = 0.974; d = -0.30 [95% CI -0.81 to 0.21]), the therapist-guided ICBT group (B = -2.54, SE = 0.87, p = 0.10; d = -0.68 [95% CI -1.13 to -0.22]), and the unguided ICBT group improved further (B = -3.55, SE = 0.97, p = 0.010; d = -0.94 [95% CI -1.45 to -0.44]).

eAppendix 9. Y-BOCS Mixed Effects Model With Quadratic Time Effect

As a sensitivity analysis, the main Y-BOCS mixed-effects model was fitted including a quadratic effect of time, along with other covariates included in the primary outcome analyses reported in the manuscript: fixed effects of group and time, their interaction, and a random intercept and slope.

The quadratic time effect was statistically significant (B = 0.02, SE = 0.001, Z = 16.57, $p < .001$), and the model had a better fit to the data compared to the model used for the primary outcome analyses (F[1] = 230.13, $p < .001$). However, as there were no repeated measures between post-treatment and 3-month follow-up, the model is likely unstable beyond the measured time-points and should not be used as a predictive model as the quadratic term skews the predictions upward.

© 2022 Lundström L et al. *JAMA Network Open.*

eFigure 3. Y-BOCS Line Graph With Quadratic Time Effect
Observed (solid lines) and estimated (dashed lines) average Y-BOCS scores from before treatment to the 3-month follow-up.

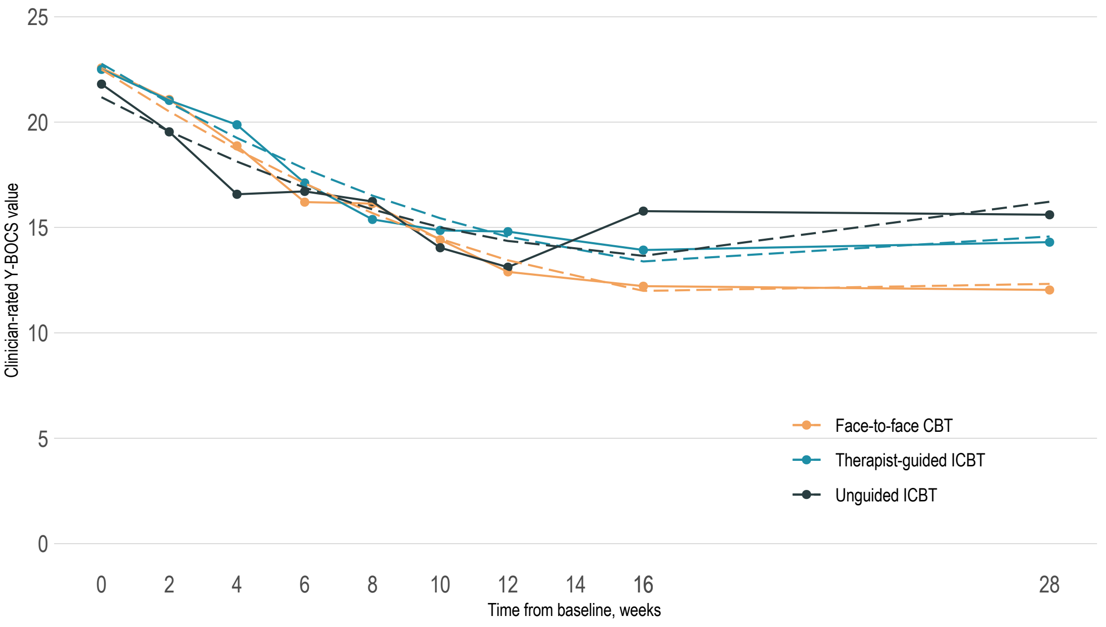

eAppendix 10. Site as a Covariate in the Main Y-BOCS Model
As a sensitivity analysis, the main Y-BOCS model was fitted including site as a covariate to investigate whether the results differed between the two treatment sites. There was no main effect of site (*B* = 2.43, SE = 1.78, *p* = 0.18) or interaction effect site x time (estimate = -0.04, SE = 0.11, *p* = 0.72).

eTable 6. Observed Means and Standard Deviations
Note that non-responders at 3-month follow-up in the two ICBT groups were offered cross-over face-to-face treatment and are included in the summary at 12-month follow-up below.

	Group		
	Face-to-face CBT	Therapist-guided ICBT	Unguided ICBT
**Y-BOCS**			
Pre-treatment	22.57 (3.78), n = 37	22.5 (3.92), n = 42	21.8 (3.04), n = 40
Week 2	21.07 (4.76), n = 27	21.03 (4.37), n = 39	19.53 (5.51), n = 30
Week 4	18.87 (5.17), n = 31	19.87 (4.62), n = 39	16.58 (5.56), n = 33
Week 6	16.2 (3.14), n = 30	17.11 (5.09), n = 35	16.71 (5.62), n = 31
Week 8	16.13 (5.27), n = 23	15.38 (4.87), n = 37	16.23 (5.17), n = 26
Week 10	14.41 (4.92), n = 29	14.86 (6.14), n = 36	14.04 (5.94), n = 26
Week 12	12.89 (4.07), n = 28	14.8 (4.54), n = 35	13.12 (5.62), n = 26
Post-treatment	12.21 (5.79), n = 33	13.93 (4.88), n = 41	15.78 (6.37), n = 36

© 2022 Lundström L et al. *JAMA Network Open.*

	Group		
	Face-to-face CBT	Therapist-guided ICBT	Unguided ICBT
3-month follow-up	12.03 (5.96), n = 31	14.3 (6.8), n = 40	15.6 (6.74), n = 35
12-month follow-up	10.8 (5.81), n = 30	11.79 (5.49), n = 38	11.9 (8.54), n = 29
**CGI-S**			
Pre-treatment	4 (0.57), n = 38	3.95 (0.58), n = 42	4 (0.39), n = 40
Post-treatment	2.81 (0.97), n = 32	2.93 (0.96), n = 41	3.22 (0.99), n = 36
3-month follow-up	2.48 (1), n = 31	2.95 (1.11), n = 40	3.11 (1.18), n = 35
12-month follow-up	2.47 (1.11), n = 30	2.61 (1.03), n = 38	2.69 (1.37), n = 29
**CGI-I**			
Post-treatment	1.97 (0.93), n = 32	2.42 (0.93), n = 40	2.81 (1.01), n = 36
3-month follow-up	1.9 (1.08), n = 31	2.55 (1.22), n = 40	2.77 (1.26), n = 35
12-month follow-up	1.9 (0.84), n = 30	2.03 (0.85), n = 38	2.21 (1.26), n = 29
**GAF**			
Pre-treatment	58.05 (4.68), n = 37	60.95 (5.92), n = 42	60.02 (4.91), n = 40
Post-treatment	64.88 (13.21), n = 32	66.38 (6.75), n = 40	64.28 (7.81), n = 36
3-month follow-up	67.87 (8.96), n = 31	65.35 (9.27), n = 40	64.89 (8.43), n = 35
12-month follow-up	68.53 (9.22), n = 30	68.08 (5.69), n = 38	69.46 (10.75), n = 28
**OCI-R**			
Pre-treatment	24.2 (9.2), n = 35	23.1 (10.22), n = 42	21.1 (12.15), n = 40
Post-treatment	12.84 (7.86), n = 32	13.37 (6.24), n = 38	12.97 (8.13), n = 35
3-month follow-up	11.52 (8), n = 31	11.64 (6.6), n = 36	12.88 (9.17), n = 32
12-month follow-up	11.54 (7.14), n = 26	9.81 (6.08), n = 27	13.9 (10.29), n = 21
**MADRS-S**			
Pre-treatment	14.66 (6.62), n = 35	12.52 (9.13), n = 42	12.44 (7.72), n = 39
Post-treatment	10.25 (7.13), n = 32	9.71 (6.94), n = 38	9.37 (6.02), n = 35
3-month follow-up	8.42 (4.12), n = 31	9.11 (7.11), n = 36	9.94 (6.69), n = 32
12-month follow-up	8.73 (5.53), n = 26	8.21 (5.62), n = 28	9.41 (8), n = 22
**EQ-5D**			

© 2022 Lundström L et al. *JAMA Network Open.*

	Group		
	Face-to-face CBT	Therapist-guided ICBT	Unguided ICBT
Pre-treatment	0.74 (0.19), n = 35	0.74 (0.24), n = 42	0.78 (0.17), n = 40
Post-treatment	0.8 (0.2), n = 32	0.82 (0.17), n = 38	0.81 (0.15), n = 35
3-month follow-up	0.86 (0.11), n = 31	0.82 (0.22), n = 36	0.8 (0.17), n = 32
12-month follow-up	0.81 (0.19), n = 26	0.86 (0.16), n = 28	0.81 (0.19), n = 22
**SDS**			
Pre-treatment	11.62 (7.56), n = 32	10.29 (7.26), n = 42	10.15 (6.44), n = 40
Post-treatment	6.38 (5.17), n = 32	6.71 (6.15), n = 38	6.97 (7.16), n = 35
3-month follow-up	4.87 (5.17), n = 31	6.36 (6.42), n = 36	7.09 (6.69), n = 32
12-month follow-up	5.15 (5.44), n = 26	4.57 (4.45), n = 28	7.05 (7.49), n = 22
**ISI**			
Pre-treatment	7.94 (5.86), n = 32	7.38 (5.86), n = 42	7.12 (5.34), n = 40
Post-treatment	6.88 (5.68), n = 32	5.45 (4.83), n = 38	6.06 (4.73), n = 35
**Y-BOCS-SR**			
Pre-treatment	20.51 (4.29), n = 35	21.14 (5.44), n = 42	20.08 (4.89), n = 40
Post-treatment	13.22 (5.54), n = 32	13.45 (5.19), n = 38	14.18 (5.81), n = 34
3-month follow-up	11.97 (4.51), n = 31	12.39 (5.88), n = 36	14 (7.07), n = 32
12-month follow-up	11.19 (4.85), n = 26	10.52 (4.52), n = 27	13.86 (7.2), n = 21

© 2022 Lundström L et al. *JAMA Network Open.*

eTable 7. Between-Group Effect Size Contrasts at Posttreatment

Between-group effect sizes were calculated using the least-squares means from mixed-effects models, using the residual standard deviation of random effects as sigma.

Between-group effect sizes were calculated using the least-squares means from mixed-effects models, using the residual standard deviation of random effects as sigma. Contrasts are presented as B – A so that positive values indicate a larger improvement for group A. Note that for EQ-5D and GAF, where an increase in total score indicates improvement, a negative effect size coefficient indicates larger improvements for group A.

	Change from pretreatment[1], *B* (SE)			Group contrasts[2]. Cohen's *d* [95% CI]		
Outcome	Face-to-face	Guided ICBT	Unguided ICBT	Face-to-face and guided ICBT	Face-to-face and unguided ICBT	Guided ICBT and unguided ICBT
OCI-R						
Post	-11.55 (1.43)	-9.27 (1.32)	-7.47 (1.37)	0.21 [-0.53 to 0.94]	0.17 [-0.57 to 0.91]	-0.03 [-0.74 to 0.68]
YBOCS-SR						
Post	-7.32 (0.98)	-7.43 (0.9)	-5.72 (0.95)	0.13 [-0.52 to 0.78]	0.29 [-0.37 to 0.96]	0.16 [-0.47 to 0.8]
MADRS-S						
Post	-4.41 (1.1)	-2.19 (1)	-2.38 (1.05)	0.02 [-0.77 to 0.8]	-0.06 [-0.86 to 0.74]	-0.08 [-0.84 to 0.69]
EQ-5D						
Post	0.06 (0.04)	0.06 (0.03)	0.02 (0.03)	0.02 [-0.59 to 0.63]	0 [-0.63 to 0.62]	-0.03 [-0.62 to 0.57]
GAF						
Post	6.69 (1.52)	5.32 (1.37)	4.25 (1.43)	0.26 [-0.34 to 0.87]	-0.06 [-0.68 to 0.55]	-0.33 [-0.92 to 0.26]
SDS						
Post	-5.43 (1.08)	-3.08 (0.96)	-2.87 (1)	0.2 [-0.54 to 0.93]	0.21 [-0.53 to 0.96]	0.02 [-0.7 to 0.73]
ISI						
Post	-0.95 (0.76)	-1.54 (0.68)	-0.84 (0.7)	-0.36 [-1.23 to 0.5]	-0.21 [-1.09 to 0.67]	0.15 [-0.69 to 0.99]

Abbreviations: CI, confidence interval; EQ-5D, EuroQol 5-dimensions; GAF, Global Assessment of Functioning; MADRS-S, Montgomery-Åsberg Depression Rating Scale Self-Rated; OCI-R, Obsessive-compulsive Inventory – Revised; SDS, Sheehan Disability Scale; YBOCS-SR, Yale-Brown Obsessive-Compulsive Scale – Self-Rated.

Explanations:

1 – Pretreatment to follow-up least squares means based on mixed effects models with a random intercept, fixed effects of time and group, and interaction effect time x group. The *B* coefficient is estimated using contrasts post – pre within each group.

2 - Between-group effect sizes were calculated using the least-squares means from mixed-effects models, using the residual standard deviation of random effects as sigma. Contrasts are presented as B – A so that positive values indicate a larger improvement for group A. Note that for EQ-5D and GAF, where an increase in total score indicates improvement, a negative effect size coefficient indicates larger improvements for group A.

© 2022 Lundström L et al. *JAMA Network Open.*

eTable 8. Long-term Effects of Secondary Outcomes

To evaluate maintenance of effects in secondary outcomes, standardized effect sizes comparing the least-squares means at 3-month and 12-month follow-up are presented below. The estimated difference is divided by the random effect residual standard deviation from the mixed-effects model. Note that non-responders at 3-month follow-up in the two ICBT groups were offered cross-over face-to-face treatment and are included in the summary at 12-month follow-up below.

Outcome	Face-to-face CBT	Therapist-guided ICBT	Unguided ICBT
OCI-R	0.06 [-0.48 to 0.6]	-0.29 [-0.81 to 0.23]	0.09 [-0.49 to 0.67]
EQ-5D	-0.28 [-0.82 to 0.26]	0.23 [-0.28 to 0.74]	0.08 [-0.49 to 0.64]
MADRS-S	-0.08 [-0.62 to 0.46]	-0.24 [-0.75 to 0.28]	-0.28 [-0.85 to 0.29]
SDS	-0.01 [-0.55 to 0.52]	-0.31 [-0.82 to 0.2]	-0.23 [-0.79 to 0.34]

eTable 9. Long-term Effects of CGI-S and CGI-I

Long-term effects of CGI-S and CGI-I[3] were based on ordinal logistic regression and is presented in the table below. The mean class is shown, based on the average of the probability distribution of each value 1-7. Note that non-responders at 3-month follow-up in the two ICBT groups were offered cross-over face-to-face treatment and are included in the summary at 12-month follow-up below.

Outcome	Group			Group x Time interaction effect, *Z*-value (p-value)	
	Face-to-face CBT	Therapist-guided ICBT	Unguided ICBT	Face-to-face and guided ICBT	Face-to-face and unguided ICBT
CGI-S	3.50	3.61	3.76	Z = 2.0 (*p* = .904)	Z = 2.62 (*p* = .034)
CGI-I	2.93	3.05	3.13	Z = 0.51 (*p* = 864)	Z = 0.79 (*p* = .709)

eTable 10. Treatment Platform Usage

	Therapist-guided ICBT	Unguided ICBT
Total login time (hours)	Mean = 45 (SD = 54), Min = 4, Max = 309	Mean = 27 (SD = 41), Min = 1, Max = 197
Number of logins	Mean = 61 (SD = 42), Min = 11, Max = 219	Mean = 30 (SD = 29), Min = 3, Max = 137
Number of sent messages	Mean = 26 (SD = 12) Min = 4, Max = 49	NA
Number of received messages	Mean = 26 (SD = 8), Min = 11, Max = 41	NA

© 2022 Lundström L et al. *JAMA Network Open.*

eTable 11. Cost Tariffs

Health service	Standard cost	Unit
General practitioner	$ 257	Consultation
Company physician	$ 140	Consultation
Psychiatrist	$ 532	Consultation
Medical specialist (other)	$ 374	Consultation
District nurse	$ 86	Consultation
Psychotherapist, private practice	$ 117	Consultation
Psychotherapist, public sector	$ 242	Consultation
Counselor	$ 231	Consultation
Physiotherapist	$ 86	Consultation
Midwife	$ 160	Consultation
Alternative care	$ 70	Consultation
Home care	$ 47	Hour
Self-help group	$ 11	Hour
Note. All costs are in 2017-06-01 US Dollar, converted from the Swedish krona. Most estimates are based on official public listings in the publicly funded Swedish health care system.		

eTable 12. Detailed Costs Breakdown at Pretreatment

	Face-to-face CBT (n = 34)	Therapist-guided ICBT (n = 42)	Unguided ICBT (n = 40)
Direct medical costs	1624 (1807), 1093	2466 (3180), 1656	1513 (1502), 811
Healthcare visits	1612 (1810), 1088	2403 (3142), 1595	1491 (1495), 770
Medication	13 (20), 6	63 (135), 26	22 (35), 2
Direct non-medical costs	0 (0), 0	25 (133), 0	0 (0), 0
Indirect costs	1054 (1515), 406	2130 (3547), 357	1299 (2579), 19
Unemployment	0 (0), 0	802 (2502), 0	211 (1332), 0
Sick leave	168 (695), 0	709 (2261), 0	570 (1602), 0
Work cutback	705 (1185), 91	434 (901), 0	399 (1113), 0
Domestic	181 (427), 28	185 (393), 5	119 (268), 0
Gross total costs	2679 (2506), 1860	4622 (5954), 2559	2812 (3202), 1643
Note. Mean (SD), median.			

eTable 13. Detailed Costs Breakdown at Posttreatment

	Face-to-face CBT (n = 32)	Therapist-guided ICBT (n = 38)	Unguided ICBT (n = 35)
Direct medical costs	1214 (1717), 79	1236 (1880), 255	992 (1350), 692
Healthcare visits	1190 (1717), 0	999 (1400), 232	971 (1338), 692
Medication	24 (59), 0	237 (1271), 20	22 (38), 6
Direct non-medical costs	26 (149), 0	6 (34), 0	0 (0), 0
Indirect costs	1251 (2314), 231	1042 (1469), 408	2496 (3713), 338
Unemployment	0 (0), 0	0 (0), 0	963 (2719), 0
Sick leave	577 (1435), 0	670 (1239), 0	991 (2513), 0
Work cutback	535 (1236), 0	281 (561), 0	420 (1642), 0
Domestic	139 (368), 0	90 (230), 0	123 (258), 0
Gross total costs	2491 (3193), 1399	2283 (2578), 1663	3489 (4357), 1424
Note. Mean (SD), median.			

eTable 14. Detailed Costs Breakdown at 3-Month Follow-up

	Face-to-face CBT (n = 31)	Therapist-guided ICBT (n = 36)	Unguided ICBT (n = 31)
Direct medical costs	394 (764), 9	790 (1388), 108	755 (1354), 74
Healthcare visits	369 (745), 0	754 (1377), 0	735 (1350), 0
Medication	25 (63), 0	36 (52), 20	21 (31), 0
Direct non-medical costs	0 (0), 0	0 (0), 0	20 (113), 0
Indirect costs	820 (2059), 0	323 (632), 3	1667 (3026), 298
Unemployment	272 (1513), 0	0 (0), 0	543 (2103), 0
Sick leave	176 (630), 0	175 (546), 0	827 (2350), 0
Work cutback	300 (777), 0	110 (230), 0	222 (474), 0
Domestic	73 (186), 0	38 (94), 0	75 (135), 0
Gross total costs	1214 (2525), 60	1112 (1781), 383	2443 (3499), 801
Note. Mean (SD), median.			

eTable 15. Detailed Costs Breakdown at 12-Month Follow-up

	Face-to-face CBT (n = 24)	Therapist-guided ICBT (n = 27)	Unguided ICBT (n = 20)
Direct medical costs	609 (1024), 63	938 (1549), 86	771 (1344), 26
Healthcare visits	573 (992), 0	905 (1533), 0	738 (1295), 0
Medication	35 (72), 7	33 (55), 18	32 (77), 13
Direct non-medical costs	0 (0), 0	0 (0), 0	84 (377), 0
Indirect costs	936 (2363), 0	927 (2386), 14	1262 (2473), 0
Unemployment	702 (2378), 0	0 (0), 0	421 (1883), 0
Sick leave	116 (325), 0	542 (2161), 0	209 (597), 0
Work cutback	86 (227), 0	265 (868), 0	494 (1025), 0
Domestic	32 (60), 0	120 (453), 0	138 (344), 0
Gross total costs	1544 (2926), 708	1865 (3515), 540	2117 (3271), 916
Note. Mean (SD), median.			

© 2022 Lundström L et al. *JAMA Network Open.*

eFigure 4. Cost-effectiveness of Therapist-Guided ICBT Versus Face-to-Face CBT at Posttreatment. Effect based on proportion of patients classified as responders.

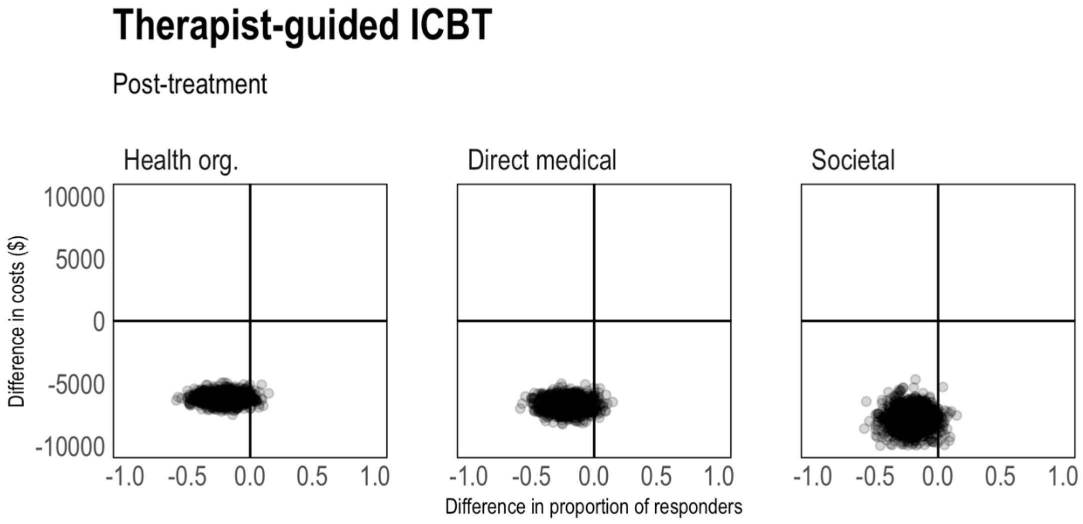

© 2022 Lundström L et al. *JAMA Network Open.*

eFigure 5. Cost-effectiveness of Therapist-Guided ICBT Versus Face-to-Face CBT at 12-Month Follow-up. Effect based on proportion of patients classified as responders.

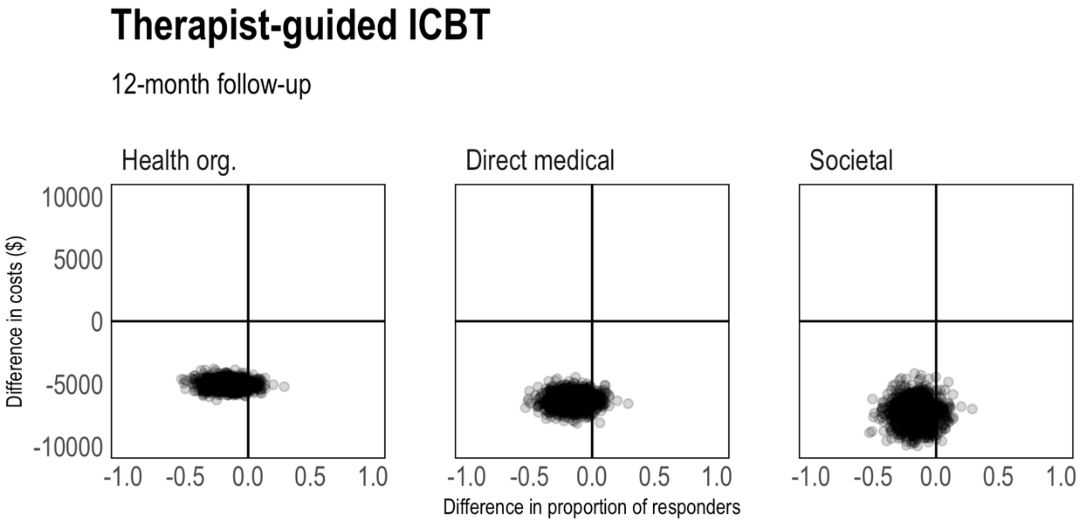

eFigure 6. Cost-effectiveness of Unguided ICBT Versus Face-to-Face CBT at Posttreatment. Effect based on proportion of patients classified as responders.

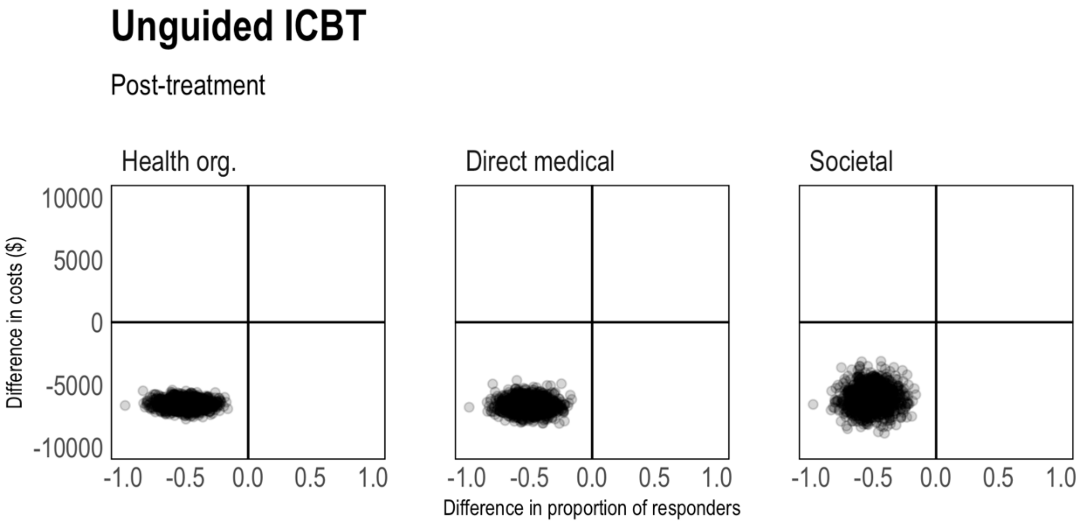

© 2022 Lundström L et al. *JAMA Network Open.*

eFigure 7. Cost-effectiveness of Unguided ICBT Versus Face-to-Face CBT at 12-Month Follow-up. Effect based on proportion of patients classified as responders.

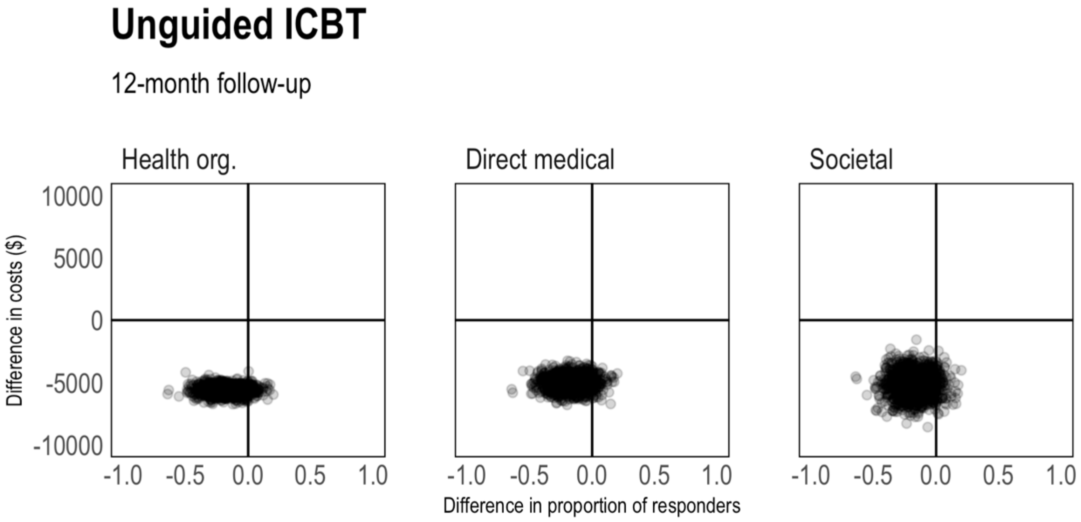

eFigure 8. Cost-Utility of Therapist-Guided ICBT Versus Face-to-Face CBT at Posttreatment. Effect based on quality-adjusted life year (QALY) change from baseline.

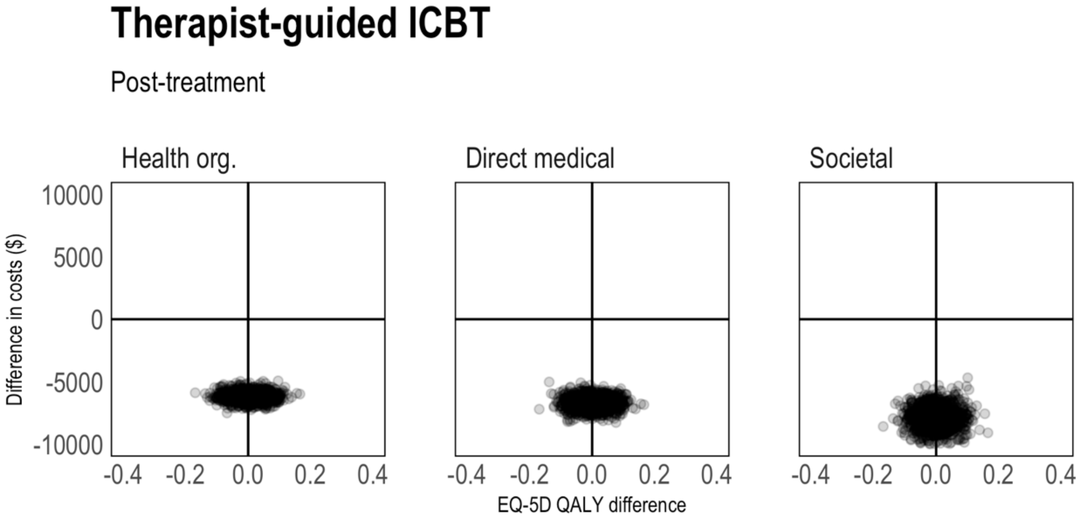

© 2022 Lundström L et al. *JAMA Network Open.*

eFigure 9. Cost-Utility of Therapist-Guided ICBT Versus Face-to-Face CBT at 3-Month Follow-up. Effect based on quality-adjusted life year (QALY) change from baseline.

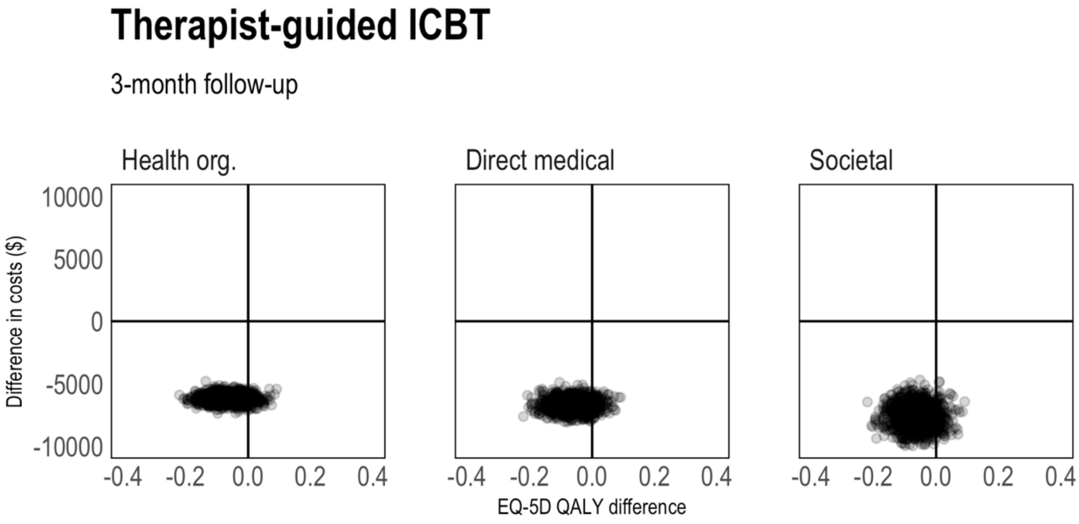

eFigure 10. Cost-Utility of Therapist-Guided ICBT Versus Face-to-Face CBT at 12-Month Follow-up. Effect based on quality-adjusted life year (QALY) change from baseline.

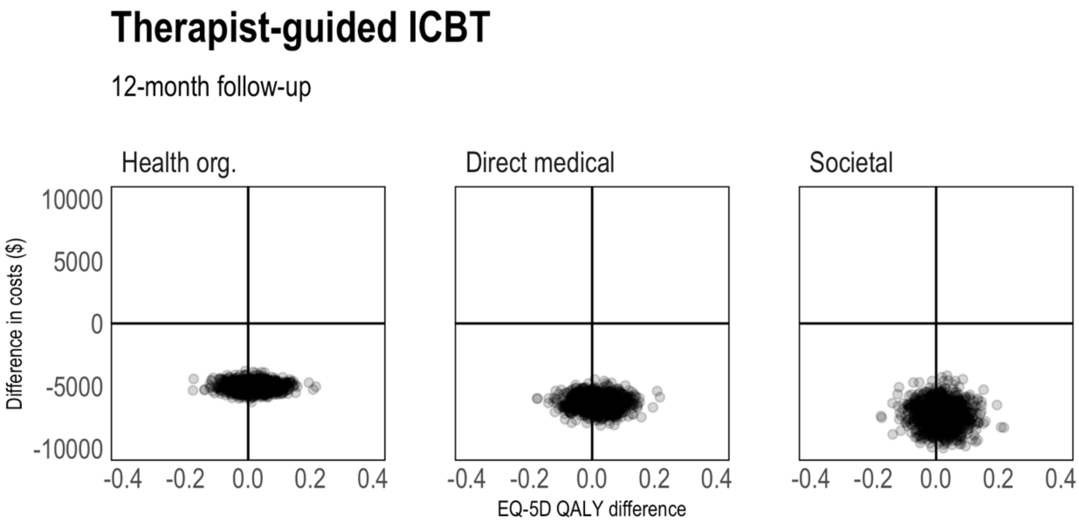

© 2022 Lundström L et al. *JAMA Network Open.*

eFigure 11. Cost-Utility of Unguided ICBT Versus Face-to-Face CBT at Posttreatment. Effect based on quality-adjusted life year (QALY) change from baseline.

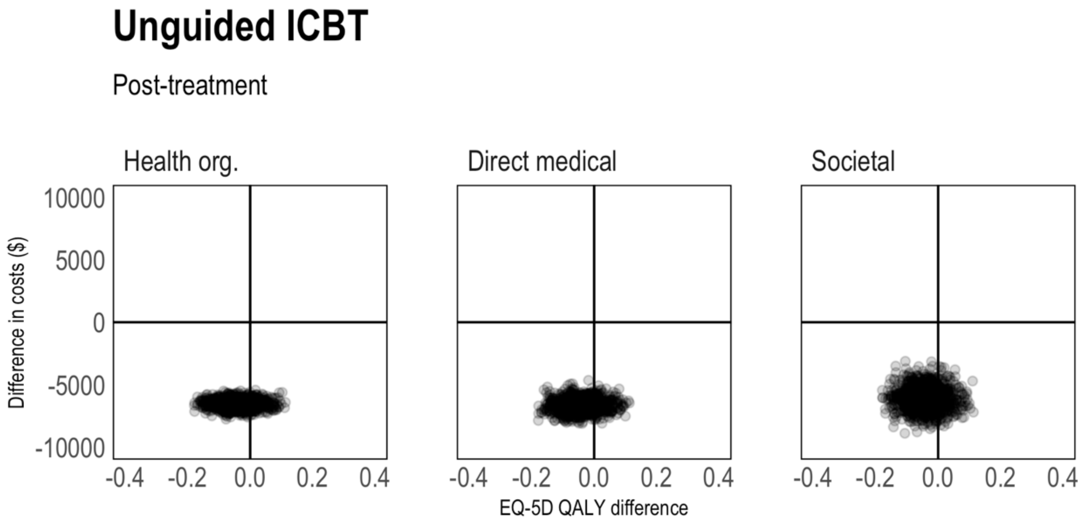

eFigure 12. Cost-Utility of Unguided ICBT Versus Face-to-Face CBT at 3-Month Follow-up. Effect based on quality-adjusted life year (QALY) change from baseline.

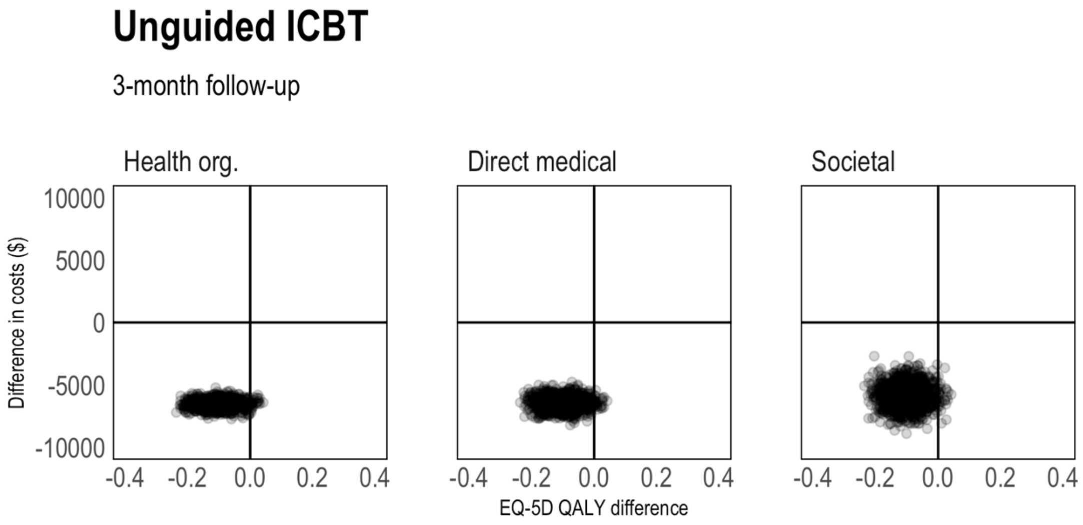

© 2022 Lundström L et al. *JAMA Network Open.*

eFigure 13. Cost-Utility of Unguided ICBT Versus Face-to-Face CBT at 12-Month Follow-up. Effect based on quality-adjusted life year (QALY) change from baseline.

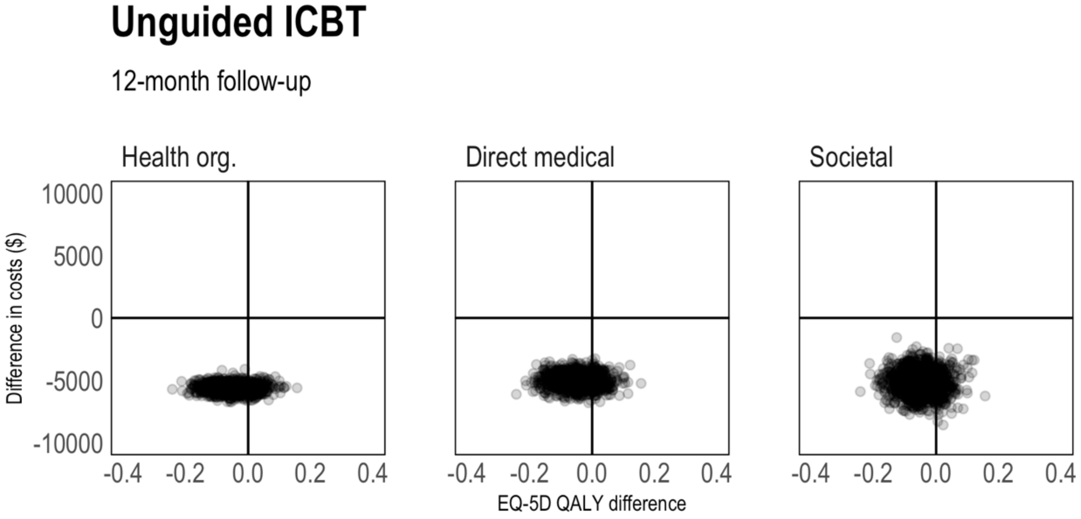

eTable 16. Treatment Costs

Treatment costs were based on the Swedish hourly tariff for psychologists of $ 242 per hour and used the following estimates: for a face-to-face session at the clinic, the 90 minutes session time were complemented by 15 minutes for administrative tasks. If a face-to-face session occurred in the patient's home or elsewhere outside the clinic, 60 minutes were added to the 90 minutes session time to account for travel time and administrative tasks (travel times up to 30 minutes one way were allowed). Therapist time in the guided ICBT group were obtained from the treatment platform, where therapist time spent on each patient is logged. For the unguided ICBT group, 60 minutes therapist time were added for platform setup and administrative tasks.

	Face-to-face CBT	Therapist-guided ICBT	Unguided ICBT
Post-treatment	6785 (878), 6763	599 (330), 577	242*
3-month follow-up	6785 (878), 6763	599 (330), 577	242
12-month follow-up	6785 (878), 6763	1705 (2513), 638	1175 (2272), 242

Note. Mean (SD), median. All costs are in 2017-06-01 US Dollars, converted from the Swedish krona. Estimates are based on the hourly tariff for psychologist visits ($242) in the publicly funded Swedish health care system.
* Since the unguided ICBT group had a fixed time of 60 minutes for platform setup and administrative tasks, there is no variation in this cost.

eAppendix 11. Costs of Crossover Treatment

Seven out of 21 non-responders in the guided ICBT group crossed over to receive additional face-to-face CBT, which increased the average treatment cost at the 12-month follow-up to $1705 (SD = $2513). The corresponding number in the unguided ICBT group was eight out of 16 non-responders, which increased the average treatment cost at the 12-month follow-up to $1175 (SD = $2272).

eAppendix 12. Response and Remission Rates

Response was defined as a reduction of 35% or more on the Y-BOCS and a CGI-I score of 1 or 2. Remission was defined as an Y-BOCS score of 12 or less and CGI-S score of 1 or 2.[34]

The number needed to treat one additional responder with face-to-face CBT compared to guided ICBT was 3.08 (95% CI 2.8 to 3.37) at the 3-month follow-up, and the corresponding number for one additional patient in remission was 3.29 (3.06 to 3.53). The number needed to treat when comparing face-to-face CBT and unguided ICBT was lower for both response (1.62, 95% CI 1.4 to 1.85) and remission (2.87, 95% CI 2.65 to 3.09), indicating that the discrepancy in efficacy was greater in this comparison. Finally, when comparing the two ICBT treatments, the number needed to treat one additional responder with guided ICBT was 3.43 (3.18 to 3.67), and for one additional patient in remission, it was 22.11 (21.97 to 22.25).

eTable 17. Long-term Response and Remission Rates

Note that non-responders at 3-month follow-up in the two ICBT groups were offered cross-over face-to-face treatment and are included in the summary at 12-month follow-up below.

Outcome	Face-to-face CBT	Therapist-guided ICBT	Unguided ICBT
Response	19 (61%), n = 31	20 (50%), n = 40	11 (35%), n = 31
Remission	16 (53%), n = 30	12 (32%), n = 38	13 (45%), n = 29
Note. Observed values only, no missing data imputation.			

eAppendix 13. Adverse Events (AEs)

- AEs that could not be attributed to the treatment such as: cough, soared throat, eye problems, fever etc. were deleted from the data
- If a participant had experienced any type of AE at two time points or more (during treatment, post treatment, at the 3-month follow-up or at the 12-month follow-up) that participant was classified as having experienced AEs during the study period
- All AEs during treatment (w 2, 4, 6, 8, 10, 12) were classified in one category (for example if a participant experienced anxiety at week 2, 6 and 12, that participant was classified as having experienced anxiety during the treatment period)
- AEs such as anxiety, sleep disturbances, depression, increased heart rate etc. were classified as mild AEs
- AEs such as suicidal thoughts and/or ideation were classified as severe AEs

eTable 18. Adverse Events During Study Period

Adverse event during study period	F2F N (%)	ICBT N (%)	Unguided ICBT N (%)	Total AE all groups	Total possible timepoints for AEs to occur all groups (120 x 4)	Percentage of AE all groups (x/480)
Anxiety	30 (20)	43 (26)	30 (19)	103	480	0,214 = 21%
Fatigue	25 (16)	39 (23)	14 (9)	78	480	0,162 = 16%
Depressive symptoms	26 (17)	26 (15)	21 (13)	73	480	0,152 = 15%
Sleep disturbances	20 (13)	22 (13)	10 (6)	52	480	0,108 = 11%
Nervousness	11 (7)	19 (11)	11 (7)	41	480	0,085 = 9 %
Headache	11 (7)	24 (14)	10 (6)	45	480	0,093 = 9%
Irritable	10 (7)	19 (11)	10 (6)	39	480	0,081 = 8%
Concentration difficulties	12 (8)	18 (11)	8 (5)	38	480	0,079 = 8%
Pressure over the chest	13 (9)	11 (7)	10 (6)	34	480	0,070 = 7%
Increased/irregular heart beat	8 (5)	18 (11)	6 (4)	32	480	0,066 = 7%
*The 10 most common mild Adverse Events (AEs) occurring during the entire study period (during treatment, at post treatment, 3-month follow-up and 12-month follow-up) using the Safety Monitoring Uniform Report Form (SMURF).[5] AEs during treatment (w 2, 4, 6, 8, 10, 12) were classified in one category "during treatment" and to be categorized the participant's AE had to						

© 2022 Lundström L et al. *JAMA Network Open.*

occur at a minimum of three bi-weekly assessments (for example if a participant experienced anxiety at week 2, 6 and 12, that participant was classified as having experienced anxiety during the treatment period).

eTable 19. Adverse Events During Treatment

**Adverse event during treatment**	**F2f N (%)**	**ICBT N (%)**	**Unguided ICBT N (%)**	**Total AE all groups**	**Total possible timepoints for AEs all groups (120)**	**Percentage of AE all groups (x/120)**
Anxiety	10 (26)	8 (19)	12 (30)	30	120	0,25 = 25%
Depressive symptoms	7 (18)	7 (17)	6 (15)	20	120	0,166 = 17%
Stress	2 (5)	6 (14)	3 (8)	11	120	0,091 = 9%
Sleep disturbances	6 (16)	2 (5)	1 (3)	9	120	0,075 = 8%
Fatigue	2 (5)	2 (5)	3 (8)	7	120	0,058 = 6%
Headache	3 (8)	1 (3)	1 (3)	5	120	0,041 = 4%
Irritable	2 (5)	1 (3)	2 (5)	5	120	0,041 = 4%
Nervousness	1 (3)	2 (5)	1 (3)	4	120	0,033 = 3%
Concentration difficulties	1 (3)	0 (0)	1 (3)	2	120	0,016 = 2%
Increased/irregular heart beat	0 (0)	1 (3)	1 (3)	2	120	0,016 = 2%

*The 10 most common mild Adverse Events (AEs) during treatment using the Safety Monitoring Uniform Report Form (SMURF).[5] AEs during treatment (w 2, 4, 6, 8, 10, 12) were classified in one category “during treatment” and to be categorized the participant’s AE had to occur at a minimum of three bi-weekly assessments (for example if a participant experienced anxiety at week 2, 6 and 12, that participant was classified as having experienced anxiety during the treatment period).

eFigure 14. Y-BOCS Noninferiority Figure With Multiple Margins and CIs

Y-BOCS figure showing both 3- and 5-point non-inferiority margins, as well as 90% confidence intervals (thick lines) and complementary 95% confidence intervals (thin lines).
Point estimates are 2.1 [90% CI –0.4 to 4.6] for therapist-guided ICBT and 5.4 [90% CI 2.8 to 7.9] for unguided ICBT. 95% confidence intervals are –0.9 to 5.1 for therapist-guided ICBT and 2.3 to 8.4 for unguided ICBT.

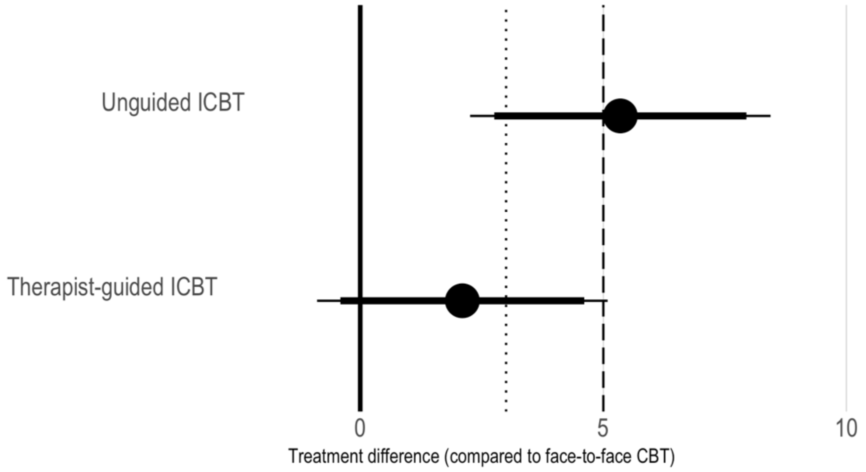

eReferences

1. First MB, Williams JBW, Karg RS, Spitzer RL. *SCID-5-CV: Structured Clinical Interview for DSM-5 Disorders: Clinician Version*. American Psychiatric Association Publishing; 2016.
2. Goodman WK, Price LH, Rasmussen SA, et al. The Yale-Brown Obsessive Compulsive Scale. I. Development, use, and reliability. *Arch Gen Psychiatry*. 1989;46(11):1006-1011. doi:10.1001/archpsyc.1989.01810110048007
3. Busner J, Targum SD. The clinical global impressions scale: applying a research tool in clinical practice. *Psychiatry Edgmont Pa Townsh*. 2007;4(7):28-37.
4. Aas IHM. Global Assessment of Functioning (GAF): properties and frontier of current knowledge. *Ann Gen Psychiatry*. 2010;9:20. doi:10.1186/1744-859X-9-20
5. Greenhill LL, Vitiello B, Fisher P, et al. Comparison of Increasingly Detailed Elicitation Methods for the Assessment of Adverse Events in Pediatric Psychopharmacology. *J Am Acad Child Adolesc Psychiatry*. 2004;43(12):1488-1496. doi:10.1097/01.chi.0000142668.29191.13
6. Simpson HB, Maher M, Page JR, Gibbons CJ, Franklin ME, Foa EB. Development of a Patient Adherence Scale for Exposure and Response Prevention Therapy. *Behav Ther*. 2010;41(1):30-37. doi:10.1016/j.beth.2008.12.002
7. Svanborg P, Åsberg M. A comparison between the Beck Depression Inventory (BDI) and the self-rating version of the Montgomery Åsberg Depression Rating Scale (MADRS). *J Affect Disord*. 2001;64(2-3):203-216. doi:10.1016/S0165-0327(00)00242-1
8. Lecrubier Y, Sheehan D, Weiller E, et al. The Mini International Neuropsychiatric Interview (MINI). A short diagnostic structured interview: reliability and validity according to the CIDI. *Eur Psychiatry*. 1997;12(5):224-231. doi:10.1016/S0924-9338(97)83296-8
9. Foa EB, Huppert JD, Leiberg S, et al. The Obsessive-Compulsive Inventory: Development and validation of a short version. *Psychol Assess*. 2002;14(4):485-496. doi:10.1037/1040-3590.14.4.485
10. EuroQol - a new facility for the measurement of health-related quality of life. *Health Policy*. 1990;16(3):199-208. doi:10.1016/0168-8510(90)90421-9
11. Bergman H, Källmén H. Alcohol use among Swedes and a psychometric evaluation of the alcohol use disorders identification test. *Alcohol Alcohol Oxf Oxfs*. 2002;37(3):245-251. doi:10.1093/alcalc/37.3.245
12. Berman AH, Wennberg, Peter, Källmén H. AUDIT & DUDIT: identifiera problem med alkohol och droger.; 2017.
13. Kroenke K, Spitzer RL, Williams JB. The PHQ-9: validity of a brief depression severity measure. *J Gen Intern Med*. 2001;16(9):606-613. doi:10.1046/j.1525-1497.2001.016009606.x
14. Sheehan DV, Harnett-Sheehan K, Raj BA. The measurement of disability. *Int Clin Psychopharmacol*. 1996;11 Suppl 3:89-95. doi:10.1097/00004850-199606003-00015
15. Bastien C. Validation of the Insomnia Severity Index as an outcome measure for insomnia research. *Sleep Med*. 2001;2(4):297-307. doi:10.1016/S1389-9457(00)00065-4
16. L R, Straten A, Tiemens B, Donker M. Manual Trimbos/iMTA Questionnaire for Costs Associated with Psychiatric Illness (TIC-P) (in Dutch). 2002;2.
17. Devilly GJ, Borkovec TD. Psychometric properties of the credibility/expectancy questionnaire. *J Behav Ther Exp Psychiatry*. 2000;31(2):73-86. doi:10.1016/S0005-7916(00)00012-4
18. Tracey TJ, Kokotovic AM. Factor structure of the Working Alliance Inventory. *Psychol Assess J Consult Clin Psychol*. 1989;1(3):207-210. doi:10.1037/1040-3590.1.3.207
19. Federici A, Summerfeldt LJ, Harrington JL, et al. Consistency between self-report and clinician-administered versions of the Yale-Brown Obsessive–Compulsive Scale. *J Anxiety Disord*. 2010;24(7):729-733. doi:10.1016/j.janxdis.2010.05.005
20. Jones SH, Thornicroft G, Coffey M, Dunn G. A Brief Mental Health Outcome Scale: Reliability and Validity of the Global Assessment of Functioning (GAF). *Br J Psychiatry*. 1995;166(5):654-659. doi:10.1192/bjp.166.5.654
21. Abramowitz JS, Deacon BJ. Psychometric properties and construct validity of the Obsessive–Compulsive Inventory—Revised: Replication and extension with a clinical sample. *J Anxiety Disord*. 2006;20(8):1016-1035. doi:10.1016/j.janxdis.2006.03.001
22. Abramowitz J, Tolin D, Diefenbach G. Measuring Change in OCD: Sensitivity of the Obsessive-Compulsive Inventory-Revised. *J Psychopathol Behav Assess*. 2005;27(4):317-324. doi:10.1007/s10862-005-2411-y
23. Svanborg P, Åsberg M. A new self-rating scale for depression and anxiety states based on the Comprehensive Psychopathological Rating Scale. *Acta Psychiatr Scand*. 1994;89(1):21-28. doi:10.1111/j.1600-0447.1994.tb01480.x

© 2022 Lundström L et al. *JAMA Network Open.*

24. Ravens-Sieberer U, Ravens-Sieberer U, Wille N, et al. Feasibility, reliability, and validity of the EQ-5D-Y: results from a multinational study. *Qual Life Res*. 2010;19(6):887-897. doi:10.1007/s11136-010-9649-x
25. Zaider TI, Heimberg RG, Fresco DM, Schneier FR, Liebowitz MR. Evaluation of the Clinical Global Impression Scale among individuals with social anxiety disorder. *Psychol Med*. 2003;33(4):611-622. doi:10.1017/S0033291703007414
26. Kadouri A, Corruble E, Falissard B. The improved Clinical Global Impression Scale (iCGI): development and validation in depression. *BMC Psychiatry*. 2007;7(1):7. doi:10.1186/1471-244X-7-7
27. Horberg N, Kouros I, Ekselius L, Ramklint M. The Swedish version of the Sheehan Disability Scale - a valid and brief measure of functioning. *Eur J Pers Centered Healthc*. 2016;4(1):208. doi:10.5750/ejpch.v4i1.1075
28. Munder T, Wilmers F, Leonhart R, Linster HW, Barth J. Working Alliance Inventory-Short Revised (WAI-SR): psychometric properties in outpatients and inpatients. *Clin Psychol Psychother*. 2010;17(3):231-239. doi:10.1002/cpp.658
29. Foa EB, Yadin E, Lichner TK. *Exposure and Response (Ritual) Prevention for Obsessive Compulsive Disorder: Therapist Guide*. Oxford University Press; 2012. doi:10.1093/med:psych/9780195335286.001.0001
30. Andersson E, Enander J, Andrén P, et al. Internet-based cognitive behaviour therapy for obsessive–compulsive disorder: a randomized controlled trial. *Psychol Med*. 2012;42(10):2193-2203. doi:10.1017/S0033291712000244
31. Andersson E, Hedman E, Enander J, et al. D -Cycloserine vs Placebo as Adjunct to Cognitive Behavioral Therapy for Obsessive-Compulsive Disorder and Interaction With Antidepressants: A Randomized Clinical Trial. *JAMA Psychiatry*. 2015;72(7):659. doi:10.1001/jamapsychiatry.2015.0546
32. Little RJA. A Test of Missing Completely at Random for Multivariate Data with Missing Values. *J Am Stat Assoc*. 1988;83(404):1198-1202. doi:10.1080/01621459.1988.10478722
33. Kessler RC, Adler L, Ames M, et al. The World Health Organization Adult ADHD Self-Report Scale (ASRS): a short screening scale for use in the general population. *Psychol Med*. 2005;35(2):245-256. doi:10.1017/s0033291704002892
34. Mataix-Cols D, de la Cruz LF, Nordsletten AE, Lenhard F, Isomura K, Simpson HB. Towards an international expert consensus for defining treatment response, remission, recovery and relapse in obsessive-compulsive disorder. *World Psychiatry*. 2016;15(1):80-81. doi:10.1002/wps.20299
35. Barber JP. Development of the Cognitive Therapy Adherence and Competence Scale. *Psychother Res*. 2003;13(2):205-221. doi:10.1093/ptr/kpg019
36. Drummond M. *Methods for the Economic Evaluation of Health Care Programmes*. Fourth edition. Oxford University Press; 2015.
37. Kanters TA, Bouwmans CAM, van der Linden N, Tan SS, Hakkaart-van Roijen L. Update of the Dutch manual for costing studies in health care. *PLOS ONE*. 2017;12(11):e0187477. doi:10.1371/journal.pone.0187477
38. Exchange rates. doi:10.1787/037ed317-en

© 2022 Lundström L et al. *JAMA Network Open.*
