# Supplementary material for: Effect of Internet-Based vs Face-to-Face Cognitive Behavioral Therapy for Adults With Obsessive-Compulsive Disorder: A Randomized Clinical Trial
Source: JAMA Netw Open. 2022 Mar 14;5(3):e221967. doi: 10.1001/jamanetworkopen.2022.1967 (PMC9907343; doi:10.1001/jamanetworkopen.2022.1967)
Supplement: Supplement 3. — Data Sharing Statement [file jamanetwopen-e221967-s003.pdf]

Data Sharing Statement

Lundström. Effect of Internet-Based vs Face-to-Face Cognitive Behavioral Therapy for Adults With Obsessive-Compulsive Disorder. *JAMA Netw Open*. Published March 14, 2022. doi:10.1001/jamanetworkopen.2022.1967

Data
Data available: No

Additional Information
Explanation for why data not available: The datasets analysed during the current study are not publicly available since they contain sensitive personal identifying information and data sharing was not part of the written informed consent but are available from the corresponding author on reasonable request. The scripts used for statistical analyses and additional materials are publicly available at https://doi.org/10.17605/OSF. IO/89BZF
